# Transcriptomic data exploring the effect of agave fructans on the induction of the defense system in avocado fruit

**DOI:** 10.1371/journal.pone.0293396

**Published:** 2023-10-26

**Authors:** Esther Angélica Cuéllar-Torres, Selene Aguilera-Aguirre, Ulises Miguel López-García, Miguel Ángel Hernández-Oñate, Efigenia Montalvo-González, Rosa Isela Ortiz-Basurto, Julio Vega-Arreguín, Alejandra Chacón-López

**Affiliations:** 1 Instituto Tecnológico de Tepic, Tecnológico Nacional de México, Tepic, Nayarit, México; 2 Coordinación de Tecnología de Alimentos de Origen Vegetal, Centro de Investigación en Alimentación y Desarrollo A.C., Hermosillo, Sonora, México; 3 Laboratorio de Ciencias Agrogenómicas and Laboratorio Nacional PlanTECC, Escuela Nacional de Estudios Superiores, Universidad Nacional Autónoma de México, León, Guanajuato, México; Abdul Wali Khan University Mardan, PAKISTAN

## Abstract

The effect of 20% high degree polymerized agave fructans (HDPAF) on the induction of the defense system in avocado fruits was investigated by transcriptomic analysis at 1, 24 and 72 h after treatment, and the effect of HDPAF on respiration rate and ethylene production was also analyzed. Transcriptomic profiling revealed 5425 differentially expressed genes (DEGs), 55 of which were involved in the pathways related to plant defense response to pathogens. Key genes were associated with phenylpropanoid biosynthesis, mitogen-activated protein signaling, plant hormone signaling, calcium ion signal decoding, and pathogenesis-related proteins. Dysregulated genes involved in ethylene biosynthesis were also identified, and the reduction in ethylene production by HDPAF was corroborated by gas chromatography, where three days of delayed peak production was observed compared to that in water-treated fruits. These results help to understand the mechanism of induction of the avocado defense system by applying HDPAF and support the application of HDPAF as an efficient postharvest treatment to extend the shelf life of the fruit.

## Introduction

Annually, postharvest losses of fruits and vegetables have reached up to 50% worldwide [1]. Several factors trigger these losses, but one of the most common is infections caused by pathogenic microorganisms, mainly phytopathogenic fungi [2]. Therefore, countries have created cooperative initiatives to reduce losses and have proposed various strategies to ensure food security [3]. The need to develop sustainable agriculture has prioritized the search for chemical compounds of natural origin, so in the last two decades, there has been significant interest in the scientific community [4]. Applying elicitors is one of the biological strategies adopted for organic agriculture and cutting the use of highly toxic microbicides. These substances are safe, non-toxic and environmentally friendly, and only micrograms of elicitors can provide long-lasting resistance against a wide range of pathogens [5, 6].

Numerous biotic elicitors have been reported to induce the plant’s innate immune system, such as carbohydrates, lipids, glycopeptides and glycoproteins, to cope with pathogen infection [7]. These elicitors act as pathogen-associated molecular patterns (PAMPs) or damage-associated molecular patterns (DAMPs) and are recognized by plant innate immune system pattern recognition receptors (PRRs) to trigger defense reactions known as PAMP-triggered immunity (PTI) [8]. Various carbohydrates and their derivatives reported to act as elicitors can activate plant defense responses and induce the plant to produce disease-resistant compounds. Carbohydrates from diverse sources can be used for different pathogens, so a series of carbohydrate pesticides can be developed for several diseases, solving the problem of ecological variation races of pathogens, which is challenging to solve by other methods [9]. In recent decades, natural products (e.g., polysaccharides and oligosaccharides) that function as elicitors, fertilizers, biostimulants and signaling molecules have aroused particular interest and are being used as novel sources of organic products in agriculture. Its use in organic agriculture induces plant defense responses, promotes plant growth, facilitates efficient nutrient uptake, increases abiotic stress tolerance and improves the shelf life and quality of food products [7, 10]. In addition, the use of polysaccharides represents a safe option to replace the use of synthetic pesticides in organic agriculture [11]; however, the structure and activity relationships of carbohydrate elicitors are poorly understood, and it is interesting to understand the molecular mechanisms involved in carbohydrate-elicitor-triggered downstream signaling.

It has been reported that high-polymerization agave fructans (HDPAF) are potential inducers of the plant defense system. Agave fructans are fructose polymers derived from sucrose with β (2→1) and β (2→6) bonds that can contain terminal or intermediate glucose [12]. Their efficacy has been evaluated in controlling diseases caused by *Phytophthora capsici* in serrano chili [13]. In addition, they have been used in avocado fruits for the control of anthracnose caused by *Colletotrichum gloeosporioides*, and it was observed an increase of the enzymatic activities of PAL, POD and PPO upon HDPAF treatment [14]. Another study reported that inulin-type fructans obtained from chicory or burdock effectively reduced gray mold symptoms caused by *Botrytis cinerea* in lettuce leaves and pointed out that fructans can act as signaling molecules an build immunity under stress conditions [10]. These studies concluded that fructans can exert a protective effect by activating defense responses; however, the molecular mechanisms involved are unclear. In recent years, transcriptome analysis has been commonly used to explore the related molecular mechanisms that control fruit postharvest diseases. In this study, we investigated the role of HDPAFs as inducers of defense mechanisms in avocado fruits and explored the genes and metabolic pathways involved in this induction by performing transcriptomic analysis.

## Materials and methods

### Fruit surface sterilization and treatment with HDPAF

The Hass avocado fruits in a state of physiological maturity (21±2% dry matter) were manually selected by experts from a local packing house (Guacamo Dely S. de R. L de C. V.) located in the municipality of Xalisco, Nayarit, Mexico (21° 43´ N, -104°90´W) and transported to the Agrobiotechnology and Electrochemical Laboratory at Tepic Technological Institute, in polypropylene boxes to avoid mechanical damage. The fruits were selected without lesions or wounds; homogeneous fruit size and weight range (170–220 g) were also considered. The fruits were superficially disinfected by immersion for 60 s in 1% sodium hypochlorite (NaOCl) solution, rinsed with water, and air-dried. Then, we followed the methodology described by [15] to collect the samples for RNA-seq. Briefly, avocado fruits at the intermediate maturity stage (5 to 7 days after harvest) were treated with 20% HDPAF or sterile distilled water as a control, and the sanitized avocado fruits were divided into six groups of three fruits each. The following treatments were applied: immersion for 60 s in sterile distilled water (control) or a HDPAF solution 20%. They were removed from the treatment to leave them for one hour in the induction period, and avocado tissue of approximately 1 cm (exocarp and mesocarp) was collected with the help of a sterile knife. Samples were taken at 1, 24, and 72 hours post-treatment (hpt) to evaluate the elicitation effect. Tissue samples were immediately frozen in liquid nitrogen and stored at -80°C until further analysis.

### Transcriptome sequencing

This study collected HDPAF-treated and control avocado samples at 1, 24 and 72 hpt; the treatments (F1, F24 and F72) and control (FC1, FC24 and FC72) samples contained three biological replicates. The total RNA extraction was performed as described by [16]. The purity and quality of the extracted total RNA were determined with the Agilent 2100 bioanalyzer spectrophotometer (S1 Fig). Library construction and transcriptome sequencing was performed at LANGEBIO (Irapuato, Mexico). The raw sequence data has been uploaded to the National Center for Biotechnology Information (NCBI) in FASTQ format, SRA repository (https://www.ncbi.nlm.nih.gov/sra/?term=PRJNA872195) under accession number SRP394641.

### Bioinformatic analysis of sequencing data

A quality check was performed on the raw data files with FASTQC software to assess the read quality filtering and clipping [17]. An additional filter was applied with Trimmomatic [18] to remove adapter sequences, eliminate reads with a quality score of less than 30, and eliminate reads with fewer than 50 nucleotides. The STAR aligner software [18] aligned the filtered reads against *Persea americana var*. drymifolia genome v3.0 (available at https://genomevolution.org/CoGe/SearchResults.pl?s=29305&p=genome). For the count of genes aligned with the reference, the featureCounts software from the Rsubread package was utilized and assigned expression values to each uniquely aligned fragment [19]. Differential gene expression analysis was performed using the Bioconductor R edgeR package [20]. False discovery rates less than 0.05 and log_2_-fold change (FC) larger than 1.0 or smaller than -1.0 were utilized for selected Differentially Expressed Genes (DEG). Genetic enrichment analyses were performed using the package erichGO to search for genetic functions and pathways overrepresented in the DGE list, and the metabolic pathways were reconstructed using the enrichKEEG of ClusterProfiler R package [21]. The scripts used for differential expression analysis, enrichment analysis and metabolic pathway assignment are available at https://github.com/Transcriptomicavocado/HDPAF-Avocado. The Pathview and Mapman tools were also used to locate the DGEs in specific metabolic pathways [22, 23].

### Respiration rate and ethylene production

The respiration rate (RR) and ethylene production rate (EPR) were measured daily for both treatments (water and 20% HDPAF) using the methodology described in Tovar et al. [24]. Fruits were randomly selected and placed individually in recipients of 1000 mL, the recipients were hermetically closed and stored at 25°C for 1 h. RR and EPR were measured by taking a 1 mL of gas in the headspace of the recipient using a syringe. The sample was then injected into a HP model 6890 gas chromatograph (San Diego, California, USA) equipped with two detectors: a CO_2_-sensitive thermal conductivity detector (TCD) and an ethylene-sensitive flame ionization detector (FID). A HP-Plot Q column (15 mm x 0.53 mm and 40 μL of film thickness) was used for gas separation. N_2_ was used as carrier gas at a 7 mL/min flow rate, H2 at 30 mL/min, and air at 400 mL/min. The injection port and the detectors were kept at 250°C. The respiration rate was expressed in mL CO_2_/kg-h, while the ethylene production rate was expressed in μL ethylene/kg-h.

## Results

### Differentially expressed genes (DGEs) in HDPAF-treated fruits

Clean reads were generated from the eighteen sample libraries, and the clean data were approximately 160.17 Gb in size and 1,458,169,132 reads. The overall sequencing error rate was 0.03%, and the average alignment percentage of the aligned clean reads from each sample assigned to the avocado genome was 85.64% (S1 Table). Clean reads were utilized for subsequent functional and expression analyses (S2 Fig).

**Fig 1:**
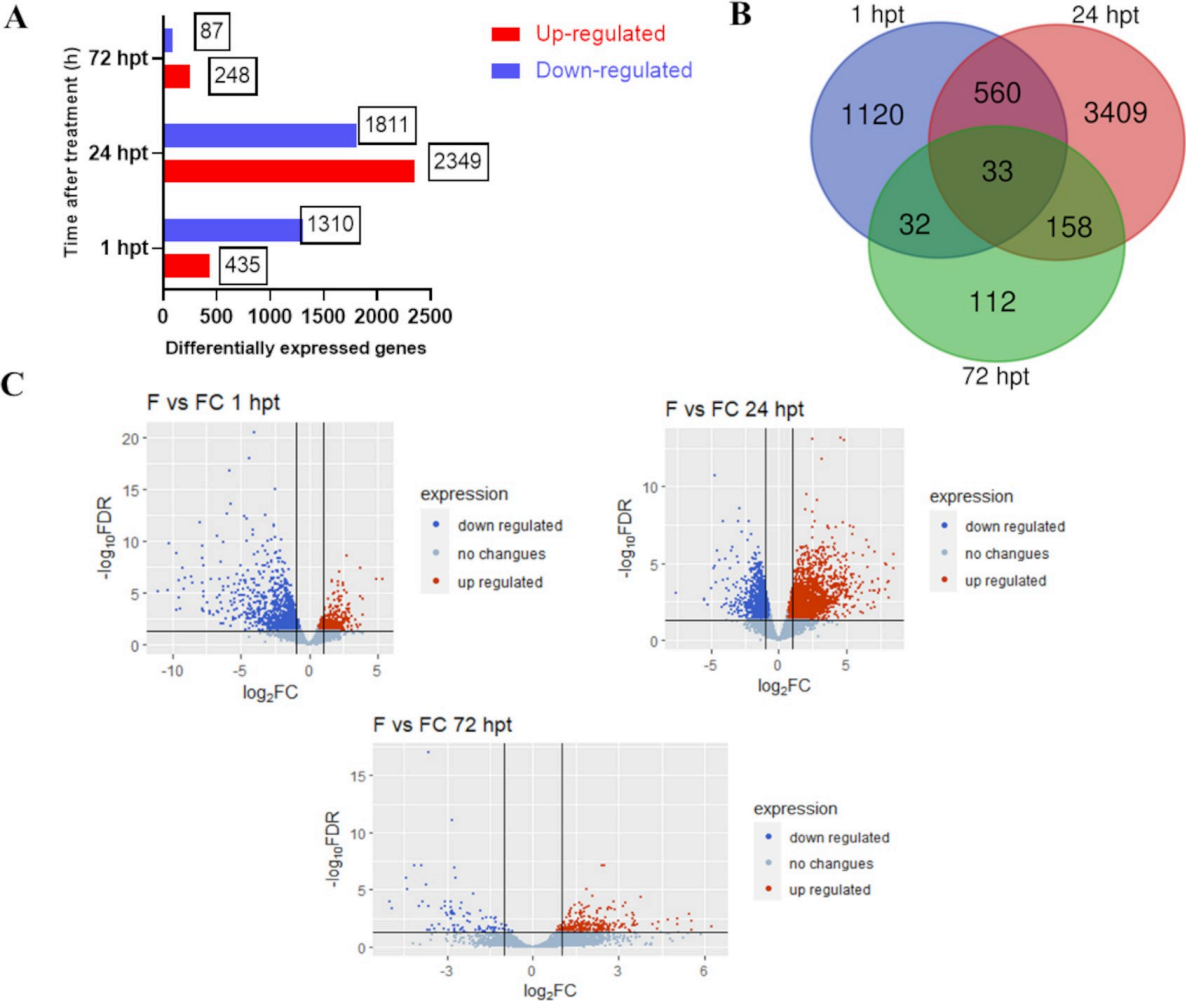
DEGs in the HDPAF-treated avocado fruit at 1, 24 and 72 hpt. (A) Bar chart and (B) Venn diagram of the number of DEGs in the three sample groups. (C) Volcano maps showing the genes with significant differential expression indicated by red dots (up-regulated) and blue dots (down-regulated); gray dots represent genes with no changes in differential expression.

In this study, 5425 genes were differentially expressed in response to 20% HDPAF treatment, and all treatments were divided into three groups, with comparison times of 1, 24 and 72 hpt, where 1745, 4160 and 335 DEGs were obtained, respectively (Fig 1A). The Venn diagram of the three comparison groups shows the number of DEGs and their overlap (Fig 1B). At 1 hpt, expression analysis revealed 1310 down-regulated and 435 up-regulated genes. The highest number of down-regulated genes was found at 24 hpt, where 2349 were up-regulated and 1811 down-regulated; at 72 hpt 248 were up-regulated, and 87 were down-regulated (Fig 1C).

### Pathway enrichment analysis for DEGs

**Fig 2:**
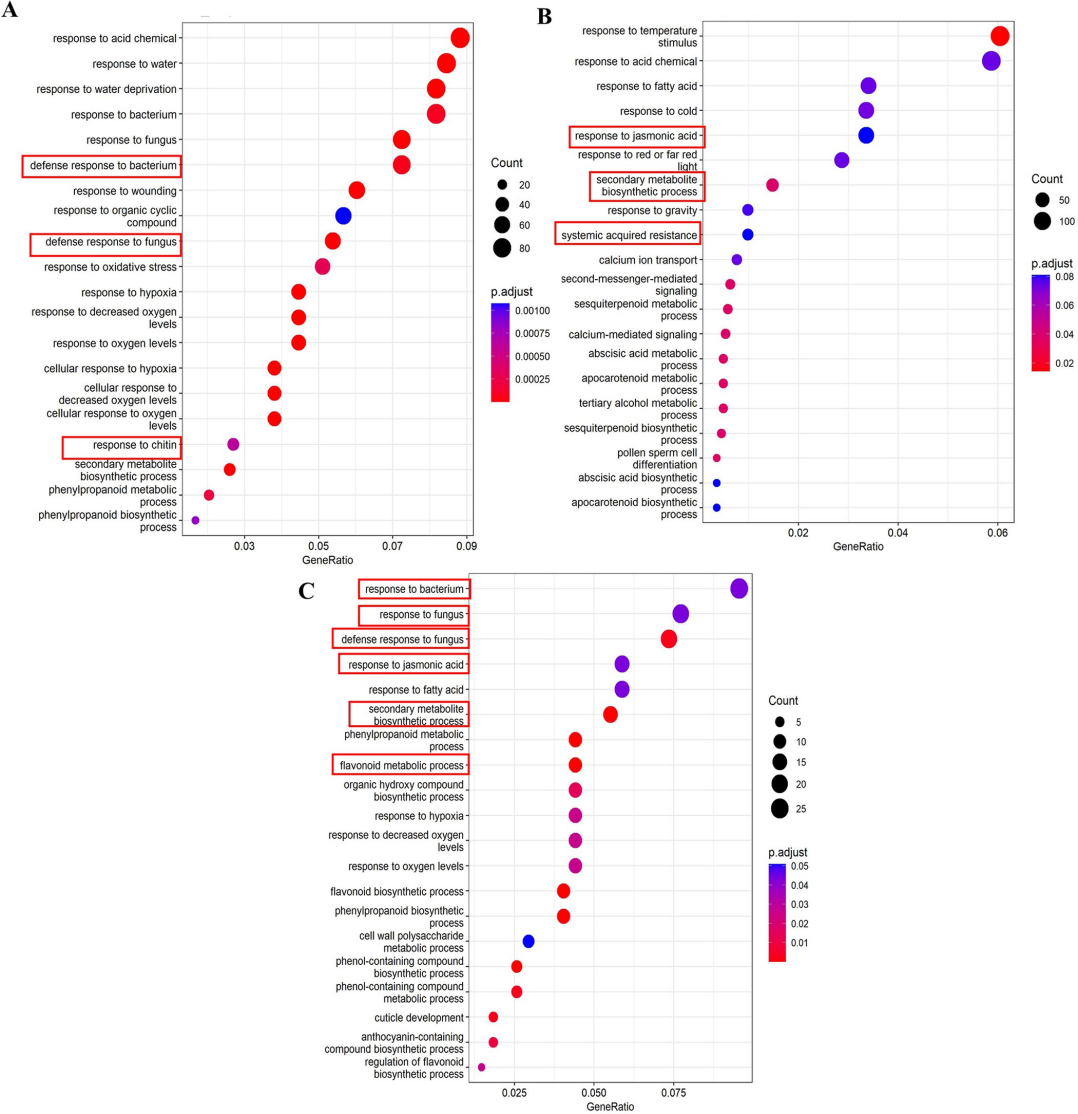
Top 20 GO terms enriched by biological processes of DEGs induced by HDPAF in avocado fruit. (A) 1 hpt, (B) 24 hpt and (C) 72 hpt. Annotation and relevant GO terms of the transcriptome of avocado samples treated with 20% HDPAF versus water, indicating the GeneRatio and P-adjusted relevance of each relevant gene belonging to the biological process category. The GO analysis included all differentially expressed genes obtained by comparing avocado samples treated with 20% HDPAF versus water, with FDR < 0.05 and logFC > |1| (5425). Gene Ratio is the GO category point size representing the number of genes in our input list that match the GO term.

As shown in Fig 2, GO term enrichment analysis revealed the main categories of biological processes related to the DGEs at 1, 24 and 72 hpt. The top 20 GO terms were selected to analyze the response of the avocado fruits to HDPAF treatment. Those at 1 hpt included defense response to bacteria, defense response to fungus, and response to chitin, among others (Fig 2A). At 24 hpt, the GO terms included the response to jasmonic acid, systemic acquired resistance, and abscisic acid metabolic process (Fig 2B). At the same time, the top 20 GO terms at 72 hpt included defense response to fungus, response to jasmonic acid, phenylpropanoid metabolic process, flavonoid biosynthetic process, and phenylpropanoid biosynthetic process (Fig 2C).

**Fig 3:**
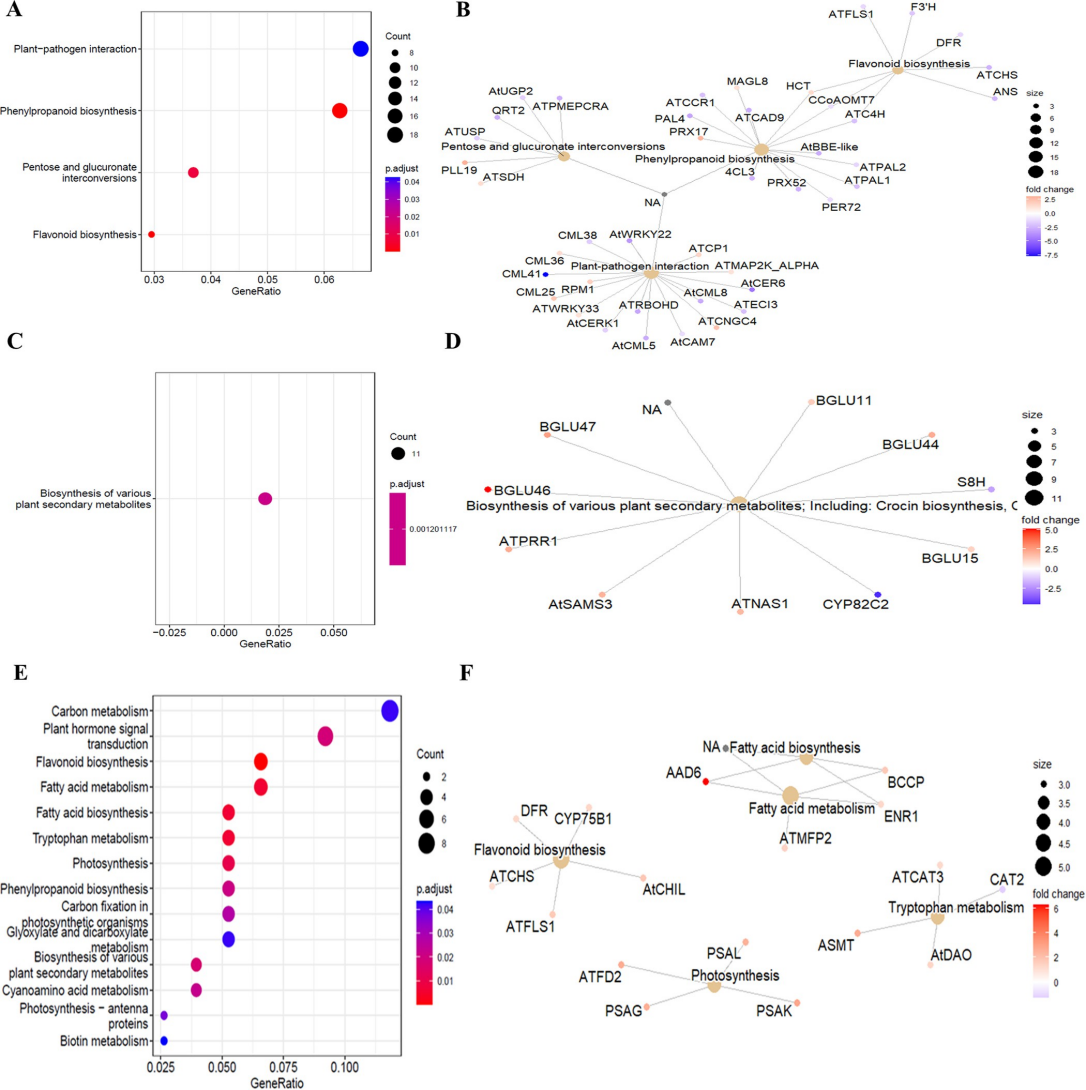
KEGG pathway analysis of DEGs in avocados treated with 20% HDPAF (F) versus water-treated avocados as control (FC).

The results of KEGG enrichment analyses at 1 hpt showed a total of 53 DEGs annotated into four pathways, as shown in Fig 3A, including “plant-pathogen interaction”, “phenylpropanoid biosynthesis”, “pentose and glucoronate interconversions”, and “flavonoid biosynthesis”. At 24 hpt, the analysis revealed “plant secondary metabolite biosynthesis” as the only enriched metabolic pathway (Fig 3C). Nevertheless, at 72 hpt, 60 DGEs were located in the pathways of “flavonoid biosynthesis”, “fatty acid metabolism and biosynthesis”, “tryptophan metabolism”, “photosynthesis”, “biosynthesis of various secondary plant metabolites”, “hormone transduction signal in plants”, “phenylpropanoid biosynthesis”, “fatty acid metabolism and biosynthesis”, “phenylpropanoid biosynthesis”, “phenylpropionic acid metabolism”, “phenylpropanoid biosynthesis”, “fatty acid metabolism and biosynthesis”, “cyanoamino acid metabolism”, “carbon fixation in photosynthetic organisms”, “photosynthesis-antenna proteins”, “carbon metabolism”, “glyoxylate and dicarboxylate metabolism”, and “biotin metabolism”(Fig 3E).

### Induction of avocado fruit defenses upon HDPAF treatment

Plant cells can activate metabolic pathways by perceiving microbial molecules as pathogen-associated molecular patterns (PAMPs). PAMPs are recognized by pattern recognition receptors (PRRs), and these PRRs can be receptor-like kinases (RLKs) or receptor-like proteins (RLPs). Induction of defense requires phosphorylation of downstream MAPK proteins; also, Ca^2+^ can regulate the respiratory burst protein oxidase homolog D (RBOHD) and activate the burst of reactive oxygen species [25]. In this sense, the results of gene enrichment at 1 hpt, genes were identified that are possibly involved in response to Ca^2+^ transduction signals (Fig 3B); among these genes, *CML5*, *CML8*, *CML38*, and *CML41* were negatively regulated, but *CML36* and *CML25* were positively regulated, which could directly influence the Ca^2+^-mediated immune response. Cyclic nucleotide-gated ion channels (CNGCs) form a large family of 20 plant members and have been implicated in Ca^2+^ signaling related to various physiological processes, such as pathogen defense, development, and thermotolerance [26]. In this sense, the *CNGC4* gene was positively regulated at 1 hpt (logFC = 2.40), and the *CNGC14* and *CNGC15* genes were positively regulated at 24 hpt (logFC = 4.54, logFC = 2.53).

In the plant-pathogen interaction pathway, transcription factors such as *WRKY22* and *WRKY33* are indirectly regulated by *MKK5* and regulate defense-related genes such as PR1. In this study, the *WRKY33* gene remained positively regulated at 1 and 24 hpt (logFC = 1.18, logFC = 1.18); however, *WRKY22* remained negatively regulated at 1 and 24 hpt (logFC = -3. 35, logFC = -1.54). Additionally, the *FLS2* gene was positively regulated at 24 hpt (logFC = 6.54), which encodes a leucine-rich receptor-like protein kinase family protein that is directly involved in the perception of bacterial flagellin by flg22 and activates the defense system against bacterial pathogens (S4 and S5 Figs). Regarding the perception by PRRs, *CERK1* is a chitin receptor that acts in the early response of plants to pathogens; in this study, *CERK1* was found to be negatively regulated at 1 hpt (logFC = -1.4) (Fig 3B). However, a gene receptor-like protein kinase 25 (CRK25) was positively regulated at 1 hpt (logFC = 4.88) (S2 Table); this gene plays an essential role in the induction of defense signaling through the recognition of carbohydrate ligands and may be involved in the perception of avocado by carbohydrate molecules such as HDPAFs.

The *RIN4* gene involved in HR and defense responses remained negatively regulated at 24 hpt (logFC = -1.29) (S2 Table and S5 Fig). Heat shock proteins such as *HSP81-1*, HSP90, and several of its co-chaperones are known as pleiotropic factors involved in the signaling pathways of multiple stress responses, including temperature, drought, and pathogen infection. *HSP81-1* was found to be positively regulated at 24 hpt (logFC = 3.25).

*CRF4*, a transcriptional regulator of genes involved in pathogenesis, remained induced at 1 and 24 hpt (logFC = 1.54, logFC = 1.82) (S2 Table). The *CER6* or *KCS6* gene is directly involved in the suppression of the HR response and defense response to the bacterial secretion system and was found to be negatively regulated at 1 hpt (logFC = -4.49), but at 24 hpt it was found to be positively regulated (logFC = 1.99) (S2 Table and S5 Fig).

### Analysis of key genes related to the secondary metabolism of avocado fruit

**Fig 4:**
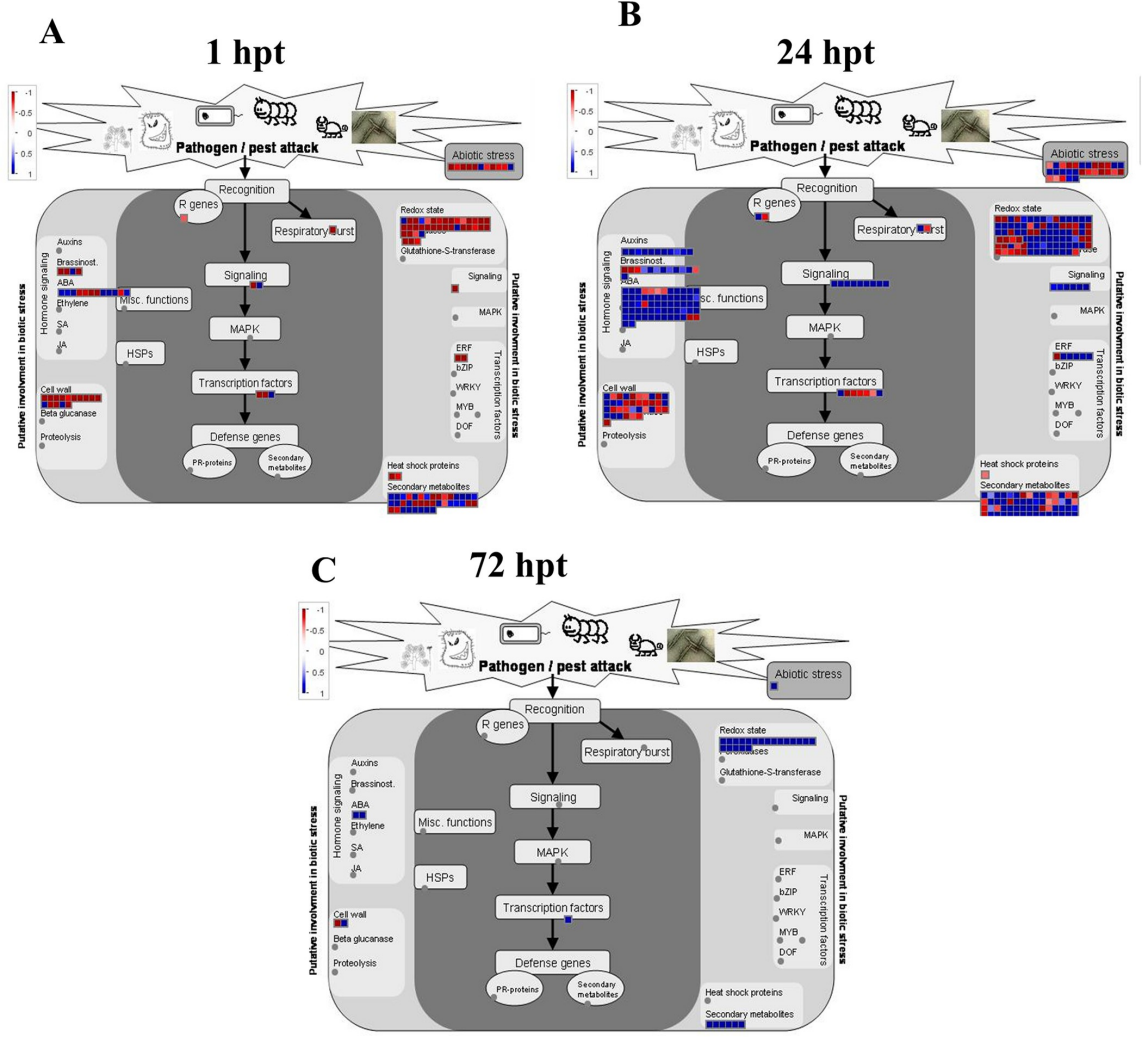
DEGs assigned to biotic and abiotic stress based on Mapman software. The map shows the dysregulated transcript’s participation in biotic and abiotic stress, redox, signaling, transcription factors, heat shock proteins, pathogenesis proteins, hormone-relative genes, and the genes implicated in the cell wall and proteolysis. Different squares indicate DEGs in FC vs. F treatments at (A) 1 hpt, (B) 24 hpt and (C) 72 hpt, where blue indicates up-regulation and red indicates down-regulation.

To activate the defense system under biotic or abiotic stress, plants synthesize secondary metabolites, including phenolic compounds and flavonoids. We used Pathview [22] and Mapman [23] tools to explore the effects of HDPAF on secondary avocado metabolism (Fig 4).

**Fig 5:**
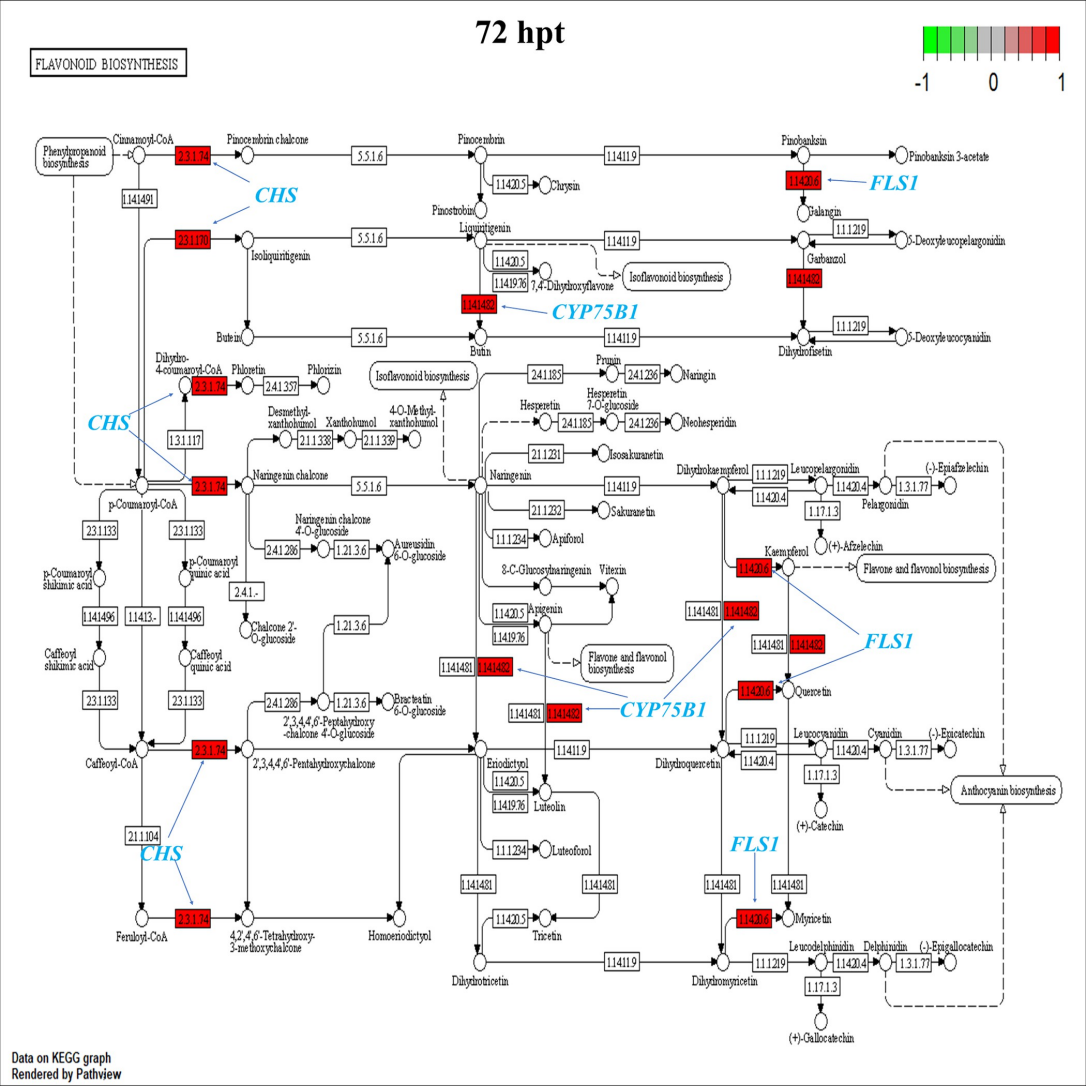
Enrichment analysis of DEGs at 72 hpt in the KEGG pathway of flavonoid biosynthesis. All colored boxes belong to genes annotated in this transcriptome result. Compared to control samples, red boxes represent DEGs with FC >1.

The results showed the highest up-regulation in the biosynthesis of secondary metabolites at 1 and 24 hpt (Fig 4A and 4B), decreasing at 72 hpt (Fig 4C). In this sense, the pathview analysis showed the negative regulation of genes involved in flavonoid biosynthesis (*C4H*, *CHS*, *HCT*, *F3H*, *FLS1* and *CCoAOMT*) at 1 and 24 hpt (S10 and S11 Figs). However, at 72 h, the *CHS*, *FLS1* and *CYP75B1* (flavonoid 3’-monooxygenase) genes were up-regulated (Fig 5) with logFC values of 1.04, 1.70 and 1.28, respectively (S2 Table).

**Fig 6:**
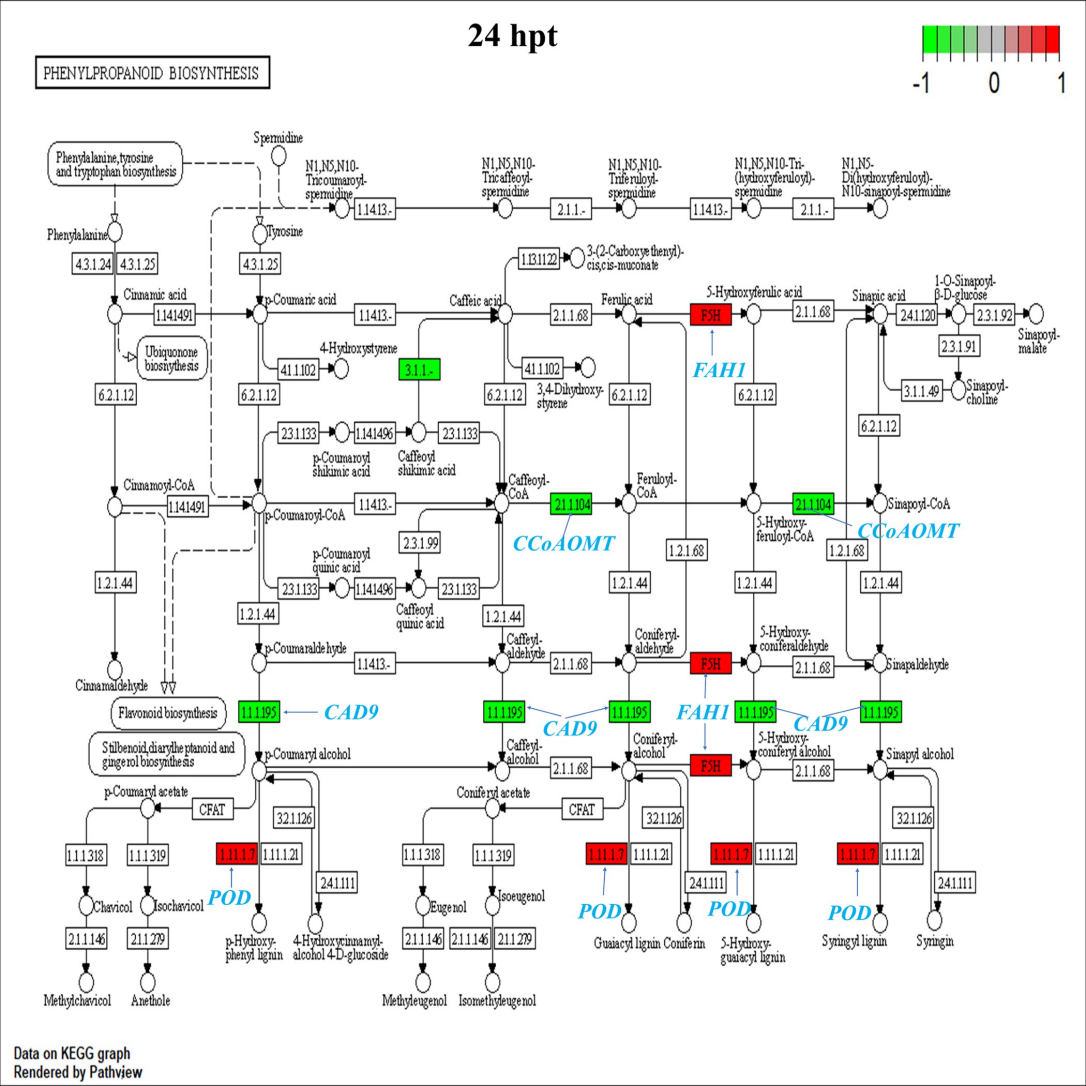
Enrichment analysis of DEGs at 24 hpt in the KEGG pathway of phenylpropanoid biosynthesis. All colored boxes belong to genes annotated in this transcriptome result. Compared to control samples, red boxes represent DEGs with FC >1, and green boxes represent DEGs with FC <1.

In this study, we found that genes negatively regulated in the phenylpropanoid biosynthesis pathway at 1 hpt, such as *PAL1*, *PAL2*, and *PAL4*, participate in the conversion of L-phenylalanine to cinnamic acid and *C4H*, which catalyzes the conversion of cinnamic acid to p-coumaric acid. Furthermore, the gene *4CL3*, which catalyzes the conversion of p-coumaric acid to p-coumaroyl-CoA, was down-regulated, whereas the *HCT* gene promoted the transformation of coumaryl-CoA to caffeoyl-CoA and was found to be positively regulated with a logFC of 4.01 (S2 Table and S12 Fig). Moreover, the *POD* genes showed negative regulation at 1 hpt and positive regulation at 24 hpt. The same was found for the *FAH1* gene coding for ferulic acid 5-hydroxylase 1, which converts coniferaldehyde to 5-hydroxyconiferaldehyde (S2 Table and Fig 6). Similarly, the expression of *DFR* increased at 72 hpt (logFC = 1.19), which directly promotes the production of apiforol and luteoforol (Fig 3B and 3F). In addition, several genes involved in the metabolism of phenylpropanoids, lignin, and terpenoids were up-regulated at 24 hpt (S16 Fig).

### MAPK signaling and hormone transduction signals in the induction of avocado defense response by HDPAF

MAPK signaling cascades mediate many biological processes that activate the response to pathogen attack during biotic and abiotic stresses and are synthesized secondary metabolites and enzymes that directly dismiss disease [27]. In HDPAF-treated avocados, transcriptome analysis revealed positive regulation of *MKK5* at 1 hpt, which is involved in late defense responses and H_2_O_2_-induced cell death (S5 Fig). Similarly, *MKK6* was positively regulated (logFC = 1.42) at 24 hpt, and could be directly involved in the regulation of the signaling cascades of the fruit in response to pathogen attack (S2 Table).

We found that genes of the response to abscisic acids (ABA), such as *PYL8/9*, *HAI3* and *ABF2* were negatively regulated (logFC = -1.35) at 1 and 24 hpt, which are directly involved in stomatal closure by transmitting ABA signals; likewise, *JAR1* was negatively regulated at 1and 24 hpt (logFC = -1.7, logFC = -1.4) this gene receives the JA signal to induce stress responses and senescence processes. Regarding the transduction signals for ET biosynthesis, the expression of *MKK5*, *EIN2* and *EBF1* genes increased at 1 hpt and decreased at 24 hpt. Additionally, *ETR1* was negatively regulated at 1 hpt. *ETR1* has been reported to trigger a defense response by transmitting ET signals, whereas *EBF1* acts downstream of *ETR1* to inhibit ET signal transmission and delay fruit ripening and senescence (S2 Table, S8 and S9 Figs).

**Fig 7:**
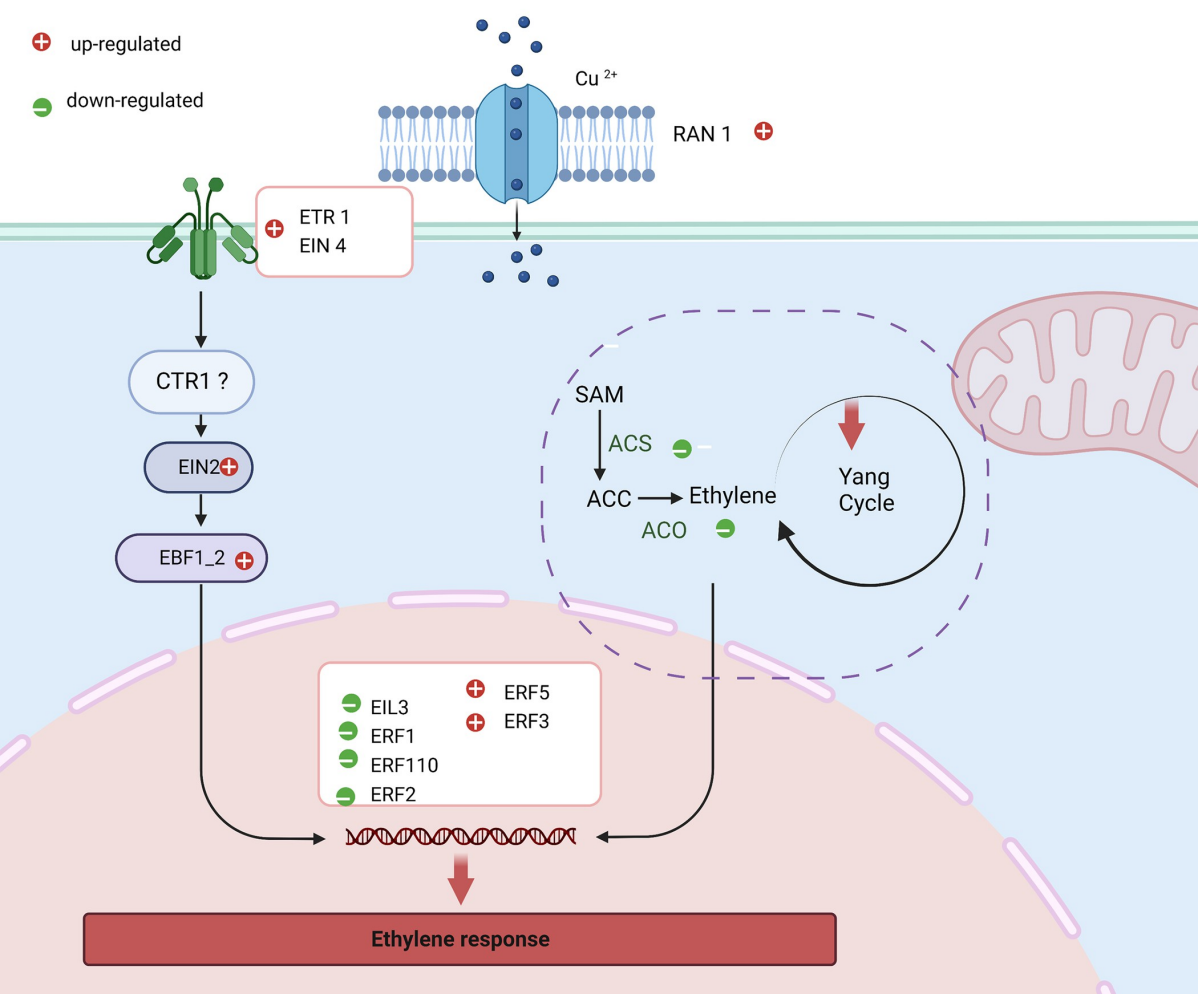
A schematic model of ethylene biosynthesis showing a few of its components affected by HDPAF in avocado fruit based on transcriptomic data obtained in the present study. Ethylene is sensed by a family of five receptors, ETR1 and EIN4, characterized by a sensor and regulatory domain [28]. In the absence of ethylene, receptors activate the kinase activity of CTR1 (constitutive triple response 1), CTR1 suppresses the downstream responses of transcription factors EIN2 and EIN3/EIL. In the presence of ethylene, receptors suppress CTR1, and EIN3/EIL transcription factors are activated, triggering ethylene signaling. When ethylene is sensed, the autocatalytic activity of ethylene forms SAM (S-adenosyl methionine) from methionine (MT), which is catalyzed by SAM synthetase, and SAM is converted to aminocyclopropane carboxylic acid (ACC) by ACC synthase and subsequently oxidized to ethylene by ACC oxidase [27]. In this study, ethylene biosynthesis was affected by HDPAF mainly through inhibition of SAM turnover via S-nitrosylation of methionine adenosyltransferase (MAT). In addition, genes encoding ACS and ACO were negatively regulated by HDPAF. Figure created with BioRender.com.

Although the pathway analysis tool did not show the regulation of *ACS*, our transcriptome analysis found two genes that code for *ACS1* at 1 and 72 hpt as negatively regulated (logFC = -3.87, logFC-2.84). Similarly, *ACO* was negatively regulated at 1 and 24 hpt (logFC = -2.18, logFC = 1.60), which could directly affect ET biosynthesis and delay the ripening process (S2 Table and Fig 7).

### Effect of HDPAF on respiration rate and ethylene production

**Fig 8:**
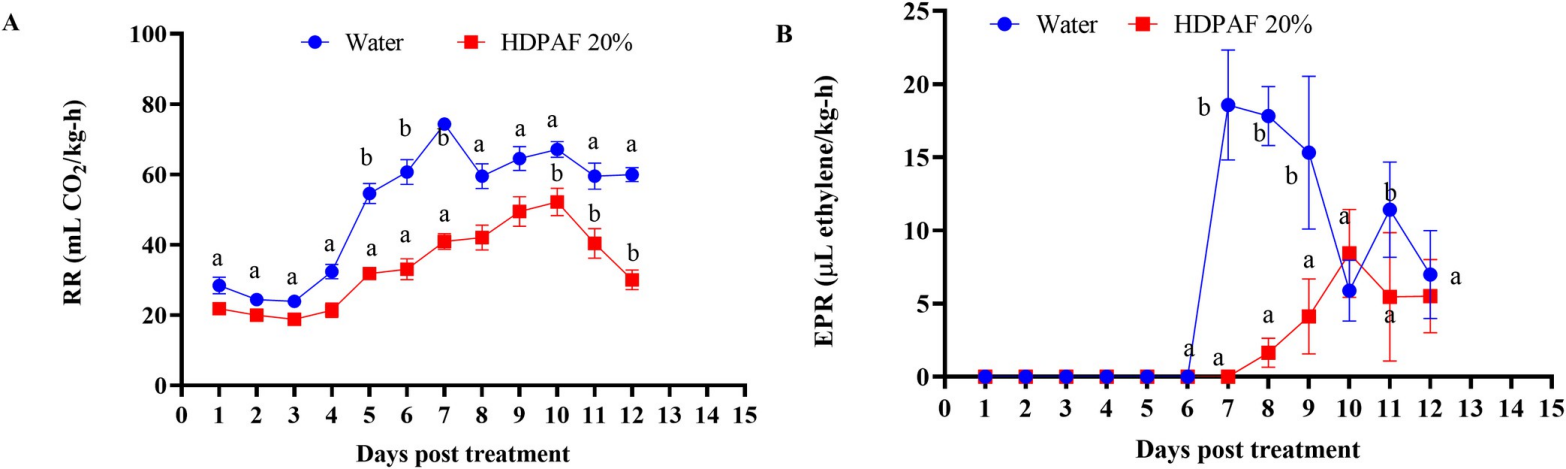
Effect of HDPAF treatment on the (A) respiration rate and (B) ethylene production during storage of avocado fruit. The fruits were treated with HDPAF at 20% w/v for 60 s and kept at room temperature (22 ± 2 C, RH 55–60%) for 12 days. Three replicates per treatment were made, and the whole experiment was performed two times. Fonts a and b represent significantly different values between the HDPAF-treated fruit and the corresponding control according to the LSD test at *p* < 0.05. Bars indicate standard errors of the mean values.

Additionally, to verify the decrease in ethylene production, the respiration rate and ethylene production were measured, where it was possible to observe the delay in the appearance of the peak of CO_2_ and ethylene production. In the avocado fruit treated with water, the climacteric peak was observed at 7 days of storage (74.32 mL CO_2_/kg-h), while the fruits treated with water presented their climacteric peak at 10 days of storage (42.04 mL CO_2_/kg-h) (Fig 8A) and with a RR lower. Similarly, for ethylene production, the fruits treated with water presented an ethylene production peak on day 7 of storage (18.56 μL ethylene/kg-h), while the fruits treated with fructans delayed their ethylene peak to day 10 of storage and with EPR minor (8.43 μL ethylene/kg-h), which meant a delay in the climacteric peak of 3 days (Fig 8B).

## Discussion

In this study, transcriptome analysis allowed us to investigate the participation of several genes involved in activating the avocado defense system following HDPAF treatment. One of the leading hypotheses about the elicitor capacity of carbohydrate polymers is that they are perceived as biotic stresses, act as PAMPs or DAMPs, and induce the immune response of plants [9]. According to the literature, only chitin, oligogalacturonides (OGAs), and β-glucans receptors are known. Chitin is the most abundant polysaccharide in nature and is present in the cell walls of fungi. When plants perceive fungal chitin, it triggers the activation of the immune system [7]. Although the receptors for other carbohydrate molecules are not known, they may be sensed through chitin, OGAs and β-glucans receptors [10] Fructans from plant or microbial sources have been tested in *Arabidopsis thaliana* during *Botrytis cinerea* infection, demonstrating their ability to activate *priming* and trigger the defense response to control the disease [29]. Because inulin-like fructans or levan fructans are accumulated in some fungi and bacteria, it has been hypothesized that these carbohydrate polymers may be perceived as pathogens and activate the defense system against abiotic and biotic stresses [7, 11]. Therefore, in this study, the activation of the avocado defense system by HDPAFs may involve the perception through the *FLS2* gene (S7 Fig) that is encoded by the receptor of the flagellin; hence, the leading hypothesis is that fructans could be perceived as a bacterium. *CERK1* gene in *Arabidopsis* and rice is considered a receptor for signaling induced by various carbohydrate elicitors [11]; in this study, we found this gene negatively regulated. Nevertheless, the positive regulation of the RECEPTOR-like protein kinase, such as *CRK25* at 1 hpt was evidenced in this transcriptome (S2 Table); as mentioned above, this gene plays an essential role in the induction of defense signaling through the recognition of carbohydrate ligands which could be directly involved in the trigger response of avocado to HDPAF treatment.

The universal second messenger involved in cellular processes, such as the defense response to pathogens through PPR signaling in plant cells, is Ca^2+^, and CNGCs mainly regulate calcium levels. Regarding calcium transport, the specific proteins that plants use to translate Ca^2+^ and ROS levels in the cytoplasm into defense transcriptional signals are calcium-binding proteins, such as CaM, CAM-like proteins (CML), Ca^2+^-dependent protein kinases (CDPK or CPK), calcineurin B-like proteins (CBL) and RBOH, as well as calmodulin-binding proteins that act as promoters of defense genes, and isochorismate synthase 1, which is an essential enzyme for SA synthesis, together these genes are important for regulating the transcription of different defense genes [30, 31]. In this study, genes such as *CNGC4*, *CNGC14* and *CNGC15* were found to be positively regulated (S2 Table, Fig 3B and S5 Fig), which could regulate the amount of Ca^2+^ needed for signaling in the avocado fruit defense response. Likewise, activating CML25 and CML36 would regulate the Ca^2+^ levels necessary to trigger the response (S2 Table). In contrast, the protein encoded by *CML* and *CNGCs* genes bound to Ca^2+^ and NO can also regulate RBOHD to induce ROS through direct binding or modification of the protein, such as CPKs [25]. Although in this study, the *CPK13* gene was negatively regulated (S5 Fig), another gene, such as *CPK29* was positively regulated (S2 Table). CPKs regulate RBOHD and play an essential role in ET biosynthesis and phosphorylation of transcription factors [30]. It has been reported that the transcription of CPKs is decreased in tobacco plants treated with BFO [32].

It has been reported that early and late defense responses to pathogens, as well as camalexin synthesis, are activated by phosphorylation of MAPK signaling cascades to induce plant defense responses, where MKK5 directly or indirectly regulates the MAPK cascade through WRKY33 that promotes transcription of defense-related genes [25]. In this study, the *MKK5* gene was positively regulated at 1 hpt (S6 Fig); similarly, transcription factors such as *WRKY33*, *WRKY41* and *WRKY44* were positively regulated at 1 and 24 hpt, which directly regulates camalexin synthesis, is activated after phosphorylation.

The *CRF4* gene is a transcriptional activator of pathogenesis-related genes, which indirectly activates the response of defense-related genes. In our transcriptome, it was found to be positively regulated at 1 and 24 hpt (S4 Fig), although in this study, genes coding for pathogenesis-related proteins such as PR1 or PR5 were not found to be deregulated. However, the transcriptome results showed genes that encode for pathogenesis-related thaumatin superfamily protein positively regulated at 24 hpt (S2 Table); moreover, in response to bacterial secretion, the positive regulation of *CER6* and *WRKY2* can induce a hypersensitive response (HR) process that has as its primary function to limit the development of pathogens. In this study, the *HSP81-1* gene was positively regulated at 24 hpt (S5 Fig), supporting the hypothesis that HDPAFs can induce HR.

Metabolites such as flavonoids, phenolic compounds, stilbenes, coumarins, terpenoids and lignins are synthesized by signaling metabolic pathways such as JA, SA or ET. Metabolites as phenolic compounds and flavonoids promote pathogen resistance in fruits by controlling pathogen development that causes membrane lipid peroxidation, which alters microbial cell membrane permeability and mitochondrial function [33]. Transcriptomic analysis showed that genes involved in the biosynthesis of these secondary metabolites, including *CHS*, *FLS1*, *CYP75B1*, *HCT*, *POD*, *FAH1*, and *DFR*, were positively regulated in avocados by HDPAF. In the flavonoid and phenylpropanoid biosynthesis pathway, the *HCT* gene is considered essential for the regulation of lignin biosynthesis and composition; in this same pathway, CHS is a crucial enzyme for flavonoid biosynthesis, *CYP75B1*, *FLS1*, together with *DFR* directly affect flavonoid accumulation in plants [34], in our study the expression *CYP75B1*, *FLS1*, *CHS* and *DFR* was regulated positively at 72 hpt in avocado by HDPAF, which supported that HDPAF enhances flavonoid accumulation in avocado.

SA biosynthesis in plants is divided into two distinct pathways, the isochorismate pathway and the phenylalanine ammonia-lyase pathway, in which the *PAL* gene was found to be negatively regulated, but the *PHYLLO* gene encoding an isochorismate synthase was found to be positively regulated at 24 hpt (S2 Table). Similar results have been reported for Kyoho grape fruits treated with chitosan [35]. SA and MAPK cascades can act upstream of each other, with some cascades triggering SA activity or SA triggering MAPK cascades, which is a complex web of interactions. Therefore, we infer that this pathway accumulates SA and acts directly for the signaling of downstream genes involved in the defense against pathogens as proteins pathogenesis-related genes such as *BGLU* and *POD* [36]. POD is responsible for oxidizing various phenyl propyl alcohols to form free radical intermediates and is an enzyme that catalyzes the last step of synthesis to form lignin polymers. The lignin forms a mechanical barrier that prevents the development of pathogens [25]. In this study, the expression of *PRXR1* (encoding a peroxidase) increased at 24 hpt in avocados by HDPAF to enhance disease resistance. Activation of the POD enzyme at 1 and 24 hpt in avocado fruit treated with HDPAF has been previously reported [14].

MAPK signaling processes are generated by a phosphorylation cascade which activates cells to induce physiological response under the perception of PRRs [37]; for example, during sensing by bacterial flagellins, the gene *FLS2* stimulates a cascade composed of MAPK1, MAPK4/5 and MAPK3/6 in leaf protoplasts of *Arabidopsis* [27], in this study the positive regulation of *FLS2* gene activated signaling cascades positively regulating MPK6, MPKK5 and MPKKK4 (S2 Table, S6 and S7 Figs).

Hormones such as SA, JA, ET and ABA act by MAPK signaling cascades that trigger responses of defense [25, 33]. During pathogen detection, these hormones operate downstream and provide another layer of regulation, affecting a multitude of developmental and response functions, including crosstalk with other hormones. JA and ET play key roles in the plant´s response to necrotrophic pathogens and herbivorous insects, and SA plays a central role in local and systemic resistance responses to biotrophic and hemibiotrophic pathogens. On the other hand, the perception of bacterial flagellin enhances the production of ET as a signaling mechanism in the induction of defense system. ET regulates the expression of defense genes and necrotroph resistance; in this sense, without ET present, EIN3 is degraded by F-box protein-mediated ubiquitination and proteasome activity. Also, ET inactivates the constitutive triple response1 (CTR1) protein, which stops the repression of EIN2 and EIN3, and up-regulates ET signaling [25]. In our transcriptome, the *EIN2* gene was positively regulated at 1 hpt. It directly activated the *EBF1* gene, activating ET absence signaling and ubiquitination-mediated proteolysis, but at 24 hpt the *ETR1* and *EIN3* genes remained negatively regulated, indicating the presence of ET and activation of defense response-related genes (S8 and S9 Figs).

In addition to the ethylene-mediated defense response, this hormone is essential for fruit ripening. The key enzymes ACS and ACO catalyzed ethylene biosynthesis begins with which is catalyzed by SAM synthetase from methionine for the production of SAM. ACS catalyzes the reaction for SAM is metabolized to 5-methylthioadenine (MTA) and incorporated into the methionine cycle to recover the sulfur atom and ACC in the presence of oxygen, ACO oxidized ACC to yield ethylene and CO_2_ [38]. The negative regulation of *ACS1*, *ACS8* and *ACO4* genes by HDPAF treatment affects ethylene and CO_2_ biosynthesis (Fig 7 and S2 Table), which directly influences the reduction of firmness loss and physiological weight loss in avocado fruit [14]. Also, it extends the shelf life of the fruit due to the delay of the climacteric peak by 3 days compared to untreated fruit (Fig 8A and 8B).

The repressing and promoting resistance responses in the presence and absence of abiotic stresses are regulated by ABA; stomata closure involves ABA signaling to regulate water loss, gas exchange, and pathogen access to the tissues [25]. In that regard, *PYL8/9*, *HAI3*, and *ABF2* genes remained negatively regulated at 1 and 24 hpt. PYL could not bind ABA in plants as *Arabidopsis* under normal conditions, and HAI3 can interact with and dephosphorylate ABF2. During the interaction between HAI3 and ABF2, the inactivation of ABF2 causes the ABA signaling pathway to be suppressed. When the ABA levels are increased, PYL interacts with and inhibits the activity of clade A HAI3, activating the signaling pathway. In this study, the negative regulation of ABA metabolic pathway genes suggests that the application of HDPAF may increase the concentration of ABA, but future studies should be conducted to test ABA concentrations in avocado fruits treated with HDPAF.

**Fig 9:**
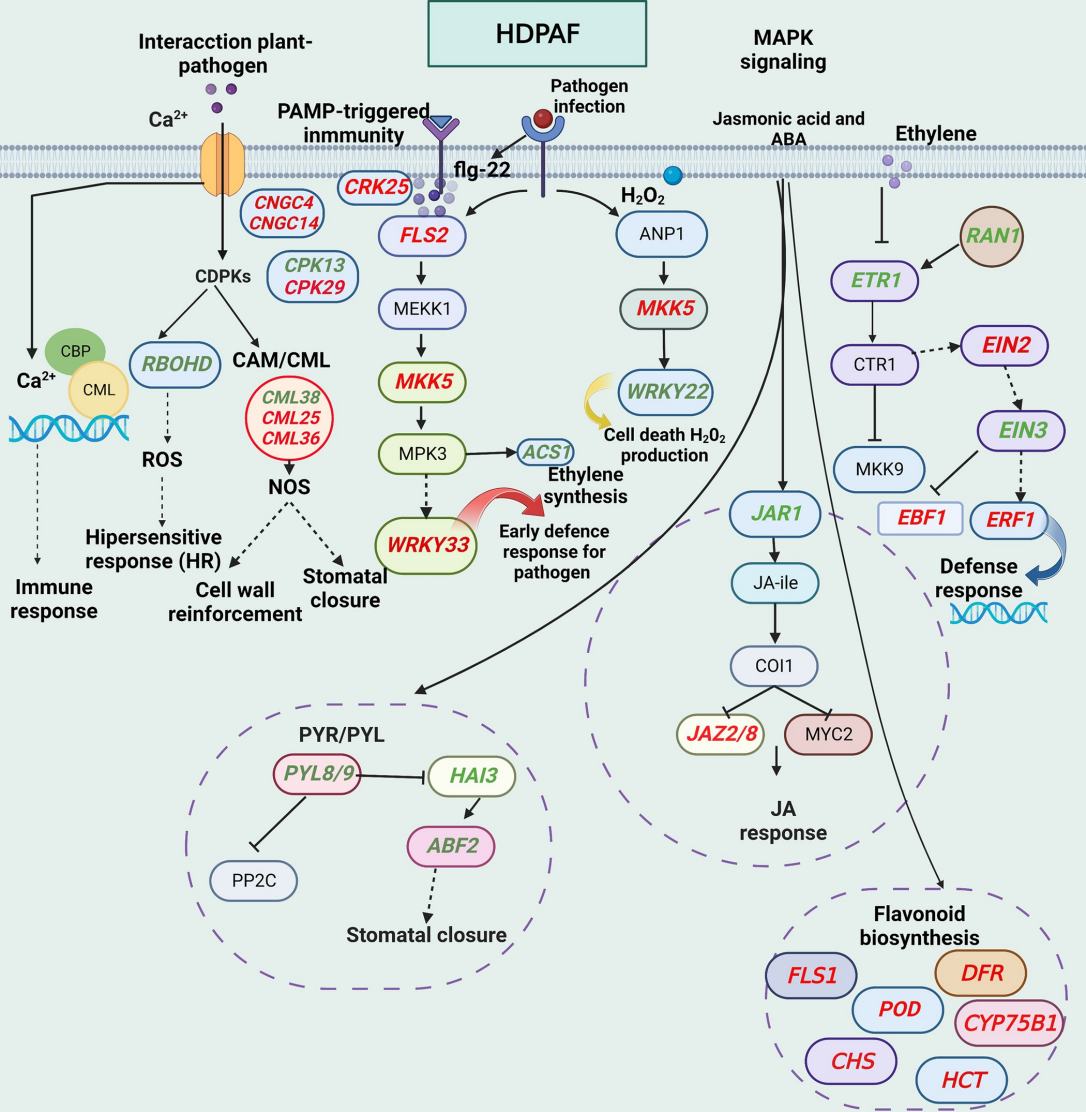
Schematic representation of the DGEs involved in metabolic pathways affected by HDPAF application in avocado fruits. Red indicates positively regulated DGEs and green indicates negatively regulated DGEs. The DGEs were located in metabolic pathways such as "plant-pathogen interaction", "calcium signaling", "flavonoid biosynthesis", "MAPK signaling", and "plant hormone-related genes". Primarily HDPAF can activate receptors necessary for the perception of bacteria, triggering MAPK signaling cascades and signal transduction pathways through hormones such as ET, JA and SA, which regulate several transcription factors essential for regulating the expression of genes related to defense mechanisms and the synthesis of secondary metabolites such as flavonoids and lignin that enhance the response of avocado plants to pathogens.

JAR1 and JAZ2/8 potentiate the defense response through AJ signaling. JA-Ile, synthesized by JA-amido synthetase (encoded by JAR1), is an endogenous disease resistance marker in plants [39]. The proteins encoded by JAZ2/8 are essential inhibitors of the JA signaling pathway and participate in the host response to *Pseudomonas syringae*. In this study, the JAR1 was negatively regulated at 1 and 24 hpt, whereas JAZ2/8 was positively regulated at 24 hpt. Based on the results of this study and the previous studies, the possible mechanisms involved in the disease resistance of avocados induced by HDPAF are shown in Fig 9.

## Conclusions

In the present study, transcriptome analysis provided relevant information regarding the genes deregulated in the central metabolic pathways involved in the defense response of avocado fruit by HDPAF application. The DGEs were located in metabolic pathways, such as "plant-pathogen interaction", "calcium signaling", "secondary metabolism", "MAPK signaling", and "plant hormone-related genes". Primarily, HDPAFs can activate receptors necessary for the perception of both bacterial and fungal pathogens, triggering MAPK signaling cascades and signal transduction pathways through hormones such as ET, JA, and SA, which regulate several transcription factors essential for regulating the expression of genes related to defense mechanisms and the synthesis of secondary metabolites such as flavonoids and lignin that enhance the response of avocado plants to pathogens. In addition, HDPAF treatment negatively regulated genes involved in ethylene biosynthesis, reflecting a decrease in the respiration rate of avocados and prolonging their shelf life.

## Supporting information

S1 Fig Quality of the RNA samples by bioanalyzer. (TIF)

S2 Fig The correlation between different samples in the interaction FC vs F. Note: Pearson correlation coefficients were calculated for all gene expression levels between samples to reflect the correlation of gene expression between samples. a) Correlation analysis between samples is reflected in heat maps. The X and Y axes represented each sample. Blue color represents the correlation coefficient of 1 (the darker the color, the higher the correlation), and in red color represents samples with a low correlation of less than 0.6; b) MDS analysis, X-axis represents the clustering of samples in dimension 2; Y-axis represents the clustering of samples in dimension 2.
(TIF)

S3 Fig Heatmaps of the top 100 deregulated genes (logFC ≥ 1 and FDR ≤ 0.01) from the F vs. FC comparison for a)1 hpt b)24 hpt, c)72 hpt. The Y-axis corresponds to the genes used as input information; the X-axis shows the different conditions with their respective biological replicates. The color key represents the median centered on the log2 values of the normalized counts.
(TIF)

S4 Fig Enrichment results of DEGs at 1 hpt in the KEGG pathway of plant-pathogen interaction. All colored boxes belong to genes annotated in this transcriptome result. Compared to control samples, red boxes represent DEGs with FC >1, and green boxes represent DEGs with FC <1.
(TIF)

S5 Fig Enrichment results of differentially expressed genes at 24 hpt in the KEGG pathway of plant-pathogen interaction. All colored boxes belong to genes annotated in this transcriptome result. Compared to control samples, red boxes represent DEGs with FC >1, and green boxes represent DEGs with FC <1.
(TIF)

S6 Fig Enrichment results of differentially expressed genes at 1 hpt in the KEGG pathway of MAPK signaling pathway-plant. All colored boxes belong to genes annotated in this transcriptome result. Compared to control samples, red boxes represent DEGs with FC >1, and green boxes represent DEGs with FC <1.
(TIF)

S7 Fig Enrichment results of differentially expressed genes at 24 hpt in the KEGG pathway of MAPK signaling pathway-plant. All colored boxes belong to genes annotated in this transcriptome result. Compared to control samples, red boxes represent DEGs with FC >1, and green boxes represent DEGs with FC <1.
(TIF)

S8 Fig Enrichment results of differentially expressed genes at 1 hpt in the KEGG pathway plant hormone signal transduction. All colored boxes belong to genes annotated in this transcriptome result. Compared to control samples, red boxes represent DEGs with FC >1, and green boxes represent DEGs with FC <1.
(TIF)

S9 Fig Enrichment results of differentially expressed genes at 24 hpt in the KEGG pathway plant hormone signal transduction. All colored boxes belong to genes annotated in this transcriptome result. Compared to control samples, red boxes represent DEGs with FC >1, and green boxes represent DEGs with FC <1.
(TIF)

S10 Fig Enrichment results of differentially expressed genes at 1 hpt in the KEGG flavonoid biosynthesis. All colored boxes belong to genes annotated in this transcriptome result. Compared to control samples, red boxes represent DEGs with FC >1, and green boxes represent DEGs with FC <1.
(TIF)

S11 Fig Enrichment results of differentially expressed genes at 24 hpt in the KEGG flavonoid biosynthesis. All colored boxes belong to genes annotated in this transcriptome result. Compared to control samples, red boxes represent DEGs with FC >1, and green boxes represent DEGs with FC <1.
(TIF)

S12 Fig Enrichment results of differentially expressed genes at 1 hpt in the KEGG phenylpropanoid biosynthesis. All colored boxes belong to genes annotated in this transcriptome result. Compared to control samples, red boxes represent DEGs with FC >1, and green boxes represent DEGs with FC <1.
(TIF)

S13 Fig Enrichment results of differentially expressed genes at 72 hpt in the KEGG phenylpropanoid biosynthesis. All colored boxes belong to genes annotated in this transcriptome result. Compared to control samples, red boxes represent DEGs with FC >1, and green boxes represent DEGs with FC <1.
(TIF)

S14 Fig Enrichment results of differentially expressed genes at 1 hpt in the KEGG phenylalanine, tyrosine and tryptophan biosynthesis. All colored boxes belong to genes annotated in this transcriptome result. Compared to control samples, red boxes represent DEGs with FC >1, and green boxes represent DEGs with FC <1.
(TIF)

S15 Fig Enrichment results of differentially expressed genes at 24 hpt in the KEGG phenylalanine, tyrosine and tryptophan biosynthesis. All colored boxes belong to genes annotated in this transcriptome result. Compared to control samples, red boxes represent DEGs with FC >1, and green boxes represent DEGs with FC <1.
(TIF)

S16 Fig Common responsive genes assigned to secondary metabolism overview (A) and phenylpropanoid biosynthesis (B) based on Mapman software. The secondary metabolism pathway consisted of the genes that participated in the shikimate pathway, the Mevalonate pathway and the biosynthesis of flavonoids, phenylpropanoids, simple phenols and lignin. Different squares indicate DEGs in FCvsFC at 24 hpt comparison, where red indicates up-regulation and blue indicates down-regulation.
(TIF)

S1 Table Statistics of the transcriptome data. (PDF)

S2 Table Details of key differentially expressed genes (DEGs) involved in disease resistance of avocado fruit. (XLSX)
