# Supplementary figures and images for: Transcriptomic data exploring the effect of agave fructans on the induction of the defense system in avocado fruit
Source: PLoS One. 2023 Oct 26;18(10):e0293396. doi: 10.1371/journal.pone.0293396 (PMC10602311; doi:10.1371/journal.pone.0293396)

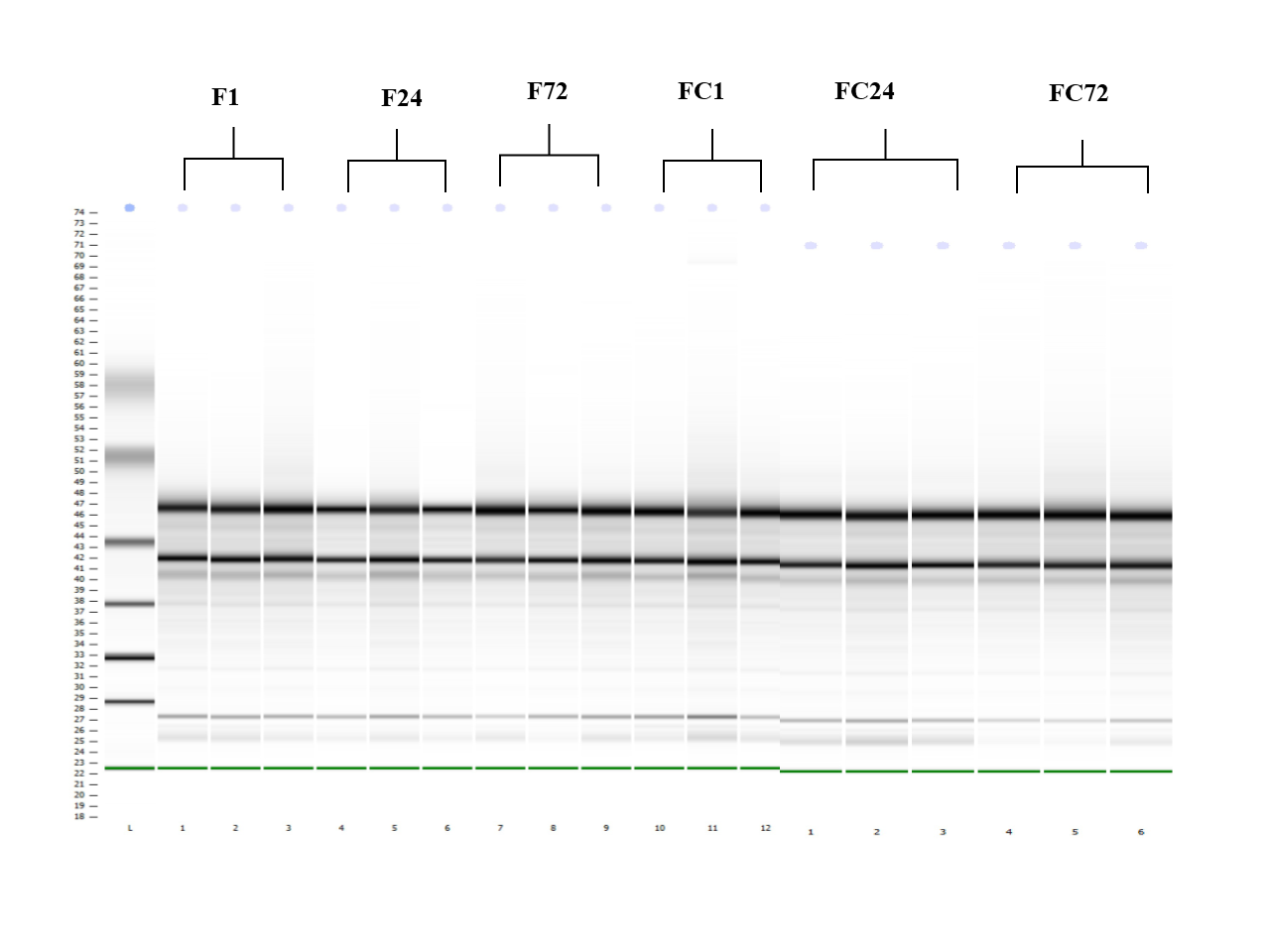

Supplement: S1 Fig — (TIF) [file pone.0293396.s001.tif]

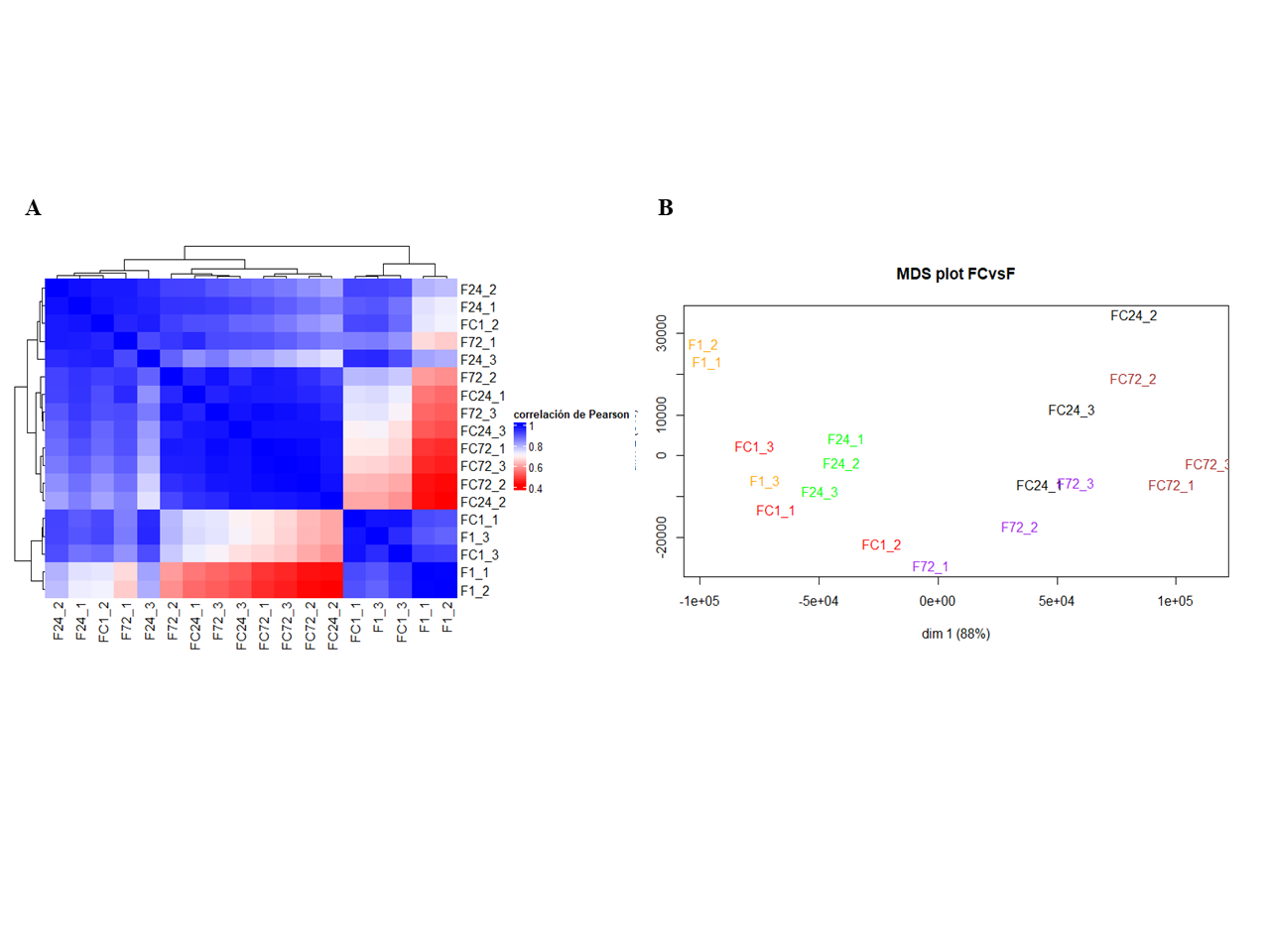

Supplement: S2 Fig — Note: Pearson correlation coefficients were calculated for all gene expression levels between samples to reflect the correlation of gene expression between samples. a) Correlation analysis between samples is reflected in heat maps. The X and Y axes represented each sample. Blue color represents the correlation coefficient of 1 (the darker the color, the higher the correlation), and in red color represents samples with a low correlation of less than 0.6; b) MDS analysis, X-axis represents the clustering of samples in dimension 2; Y-axis represents the clustering of samples in dimension 2. (TIF) [file pone.0293396.s002.tif]

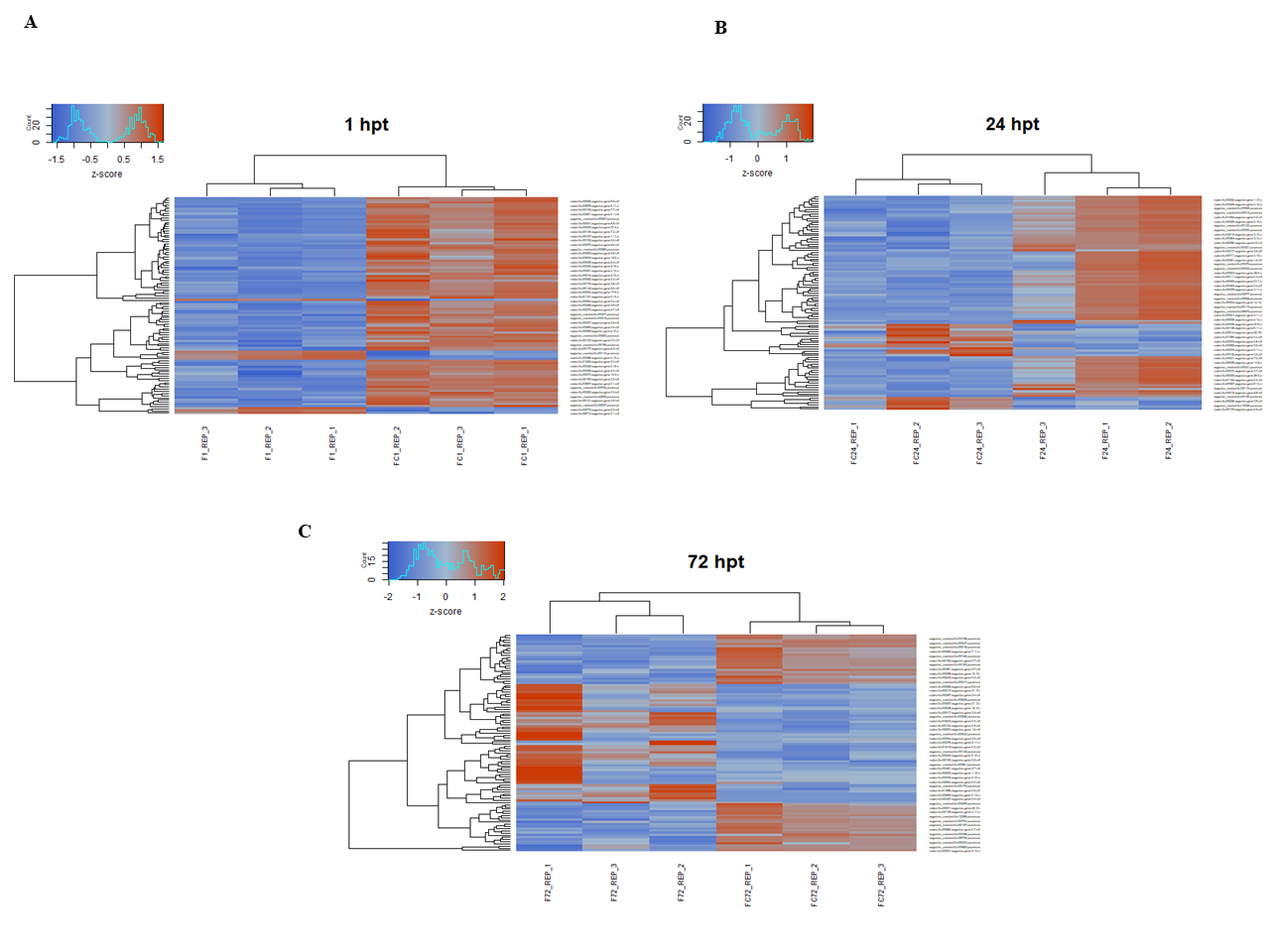

Supplement: S3 Fig — Heatmaps of the top 100 deregulated genes (logFC ≥ 1 and FDR ≤ 0.01) from the F vs. FC comparison for a)1 hpt b)24 hpt, c)72 hpt. The Y-axis corresponds to the genes used as input information; the X-axis shows the different conditions with their respective biological replicates. The color key represents the median centered on the log2 values of the normalized counts. (TIF) [file pone.0293396.s003.tif]

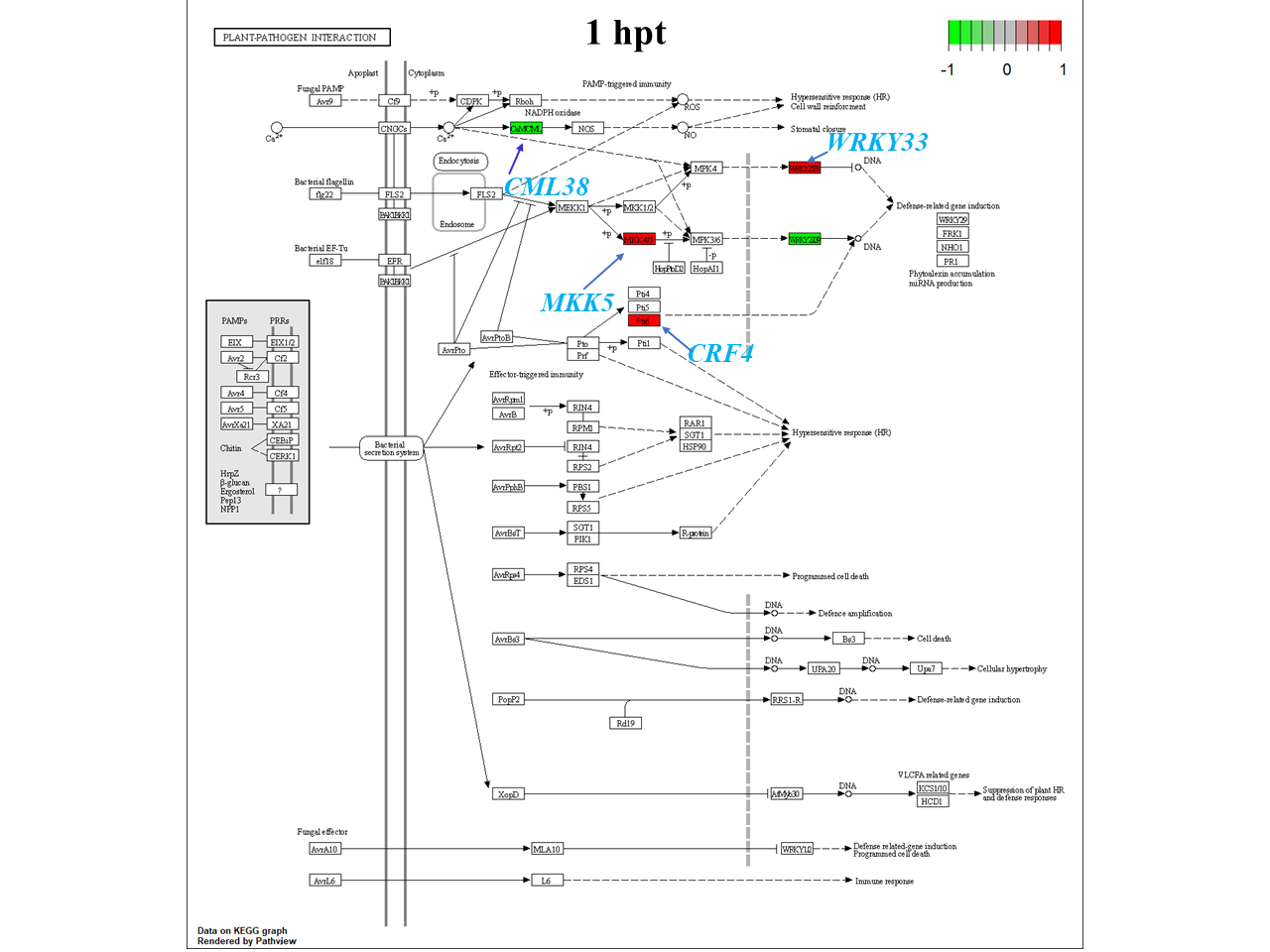

Supplement: S4 Fig — All colored boxes belong to genes annotated in this transcriptome result. Compared to control samples, red boxes represent DEGs with FC >1, and green boxes represent DEGs with FC <1. (TIF) [file pone.0293396.s004.tif]

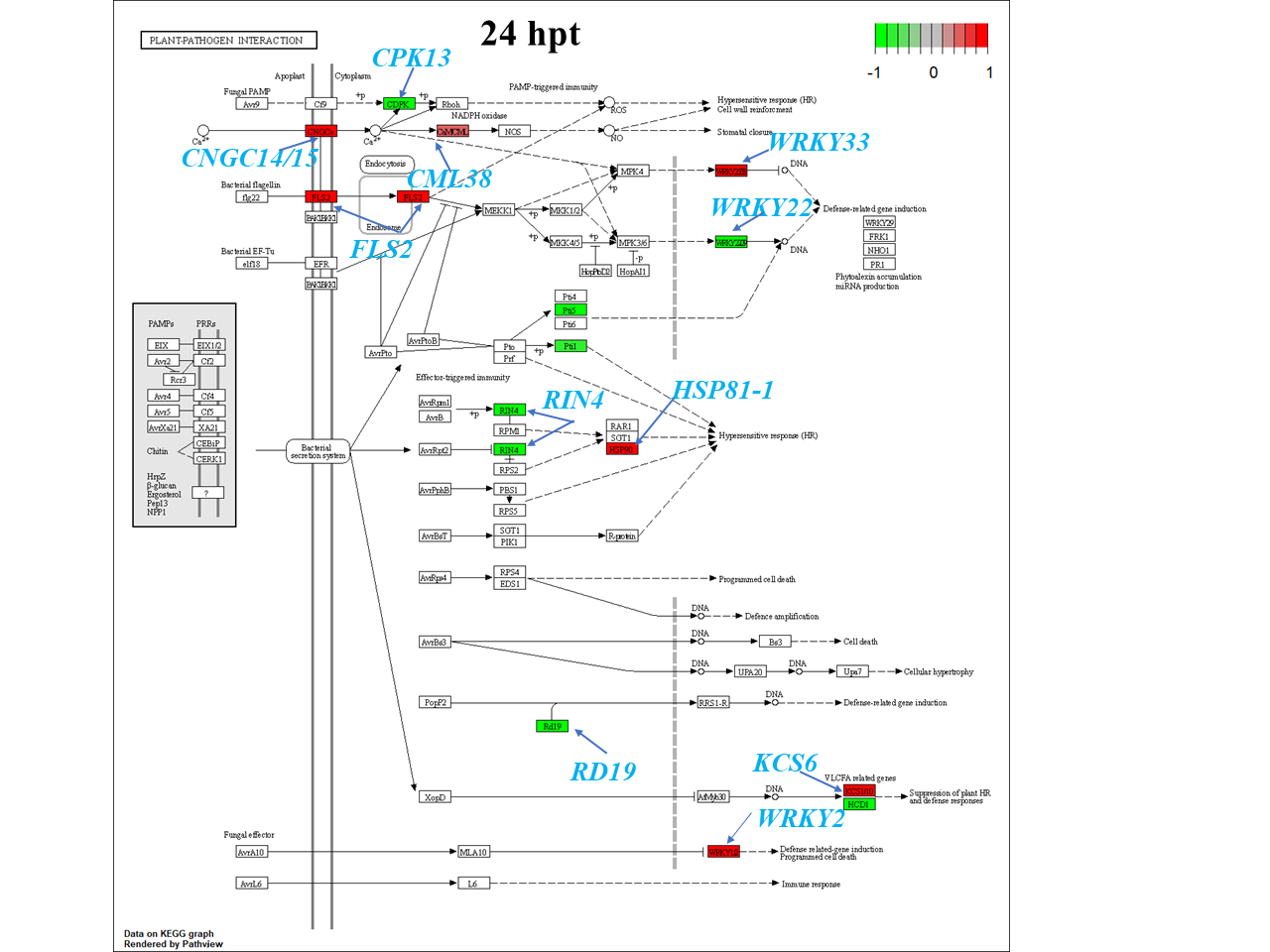

Supplement: S5 Fig — All colored boxes belong to genes annotated in this transcriptome result. Compared to control samples, red boxes represent DEGs with FC >1, and green boxes represent DEGs with FC <1. (TIF) [file pone.0293396.s005.tif]

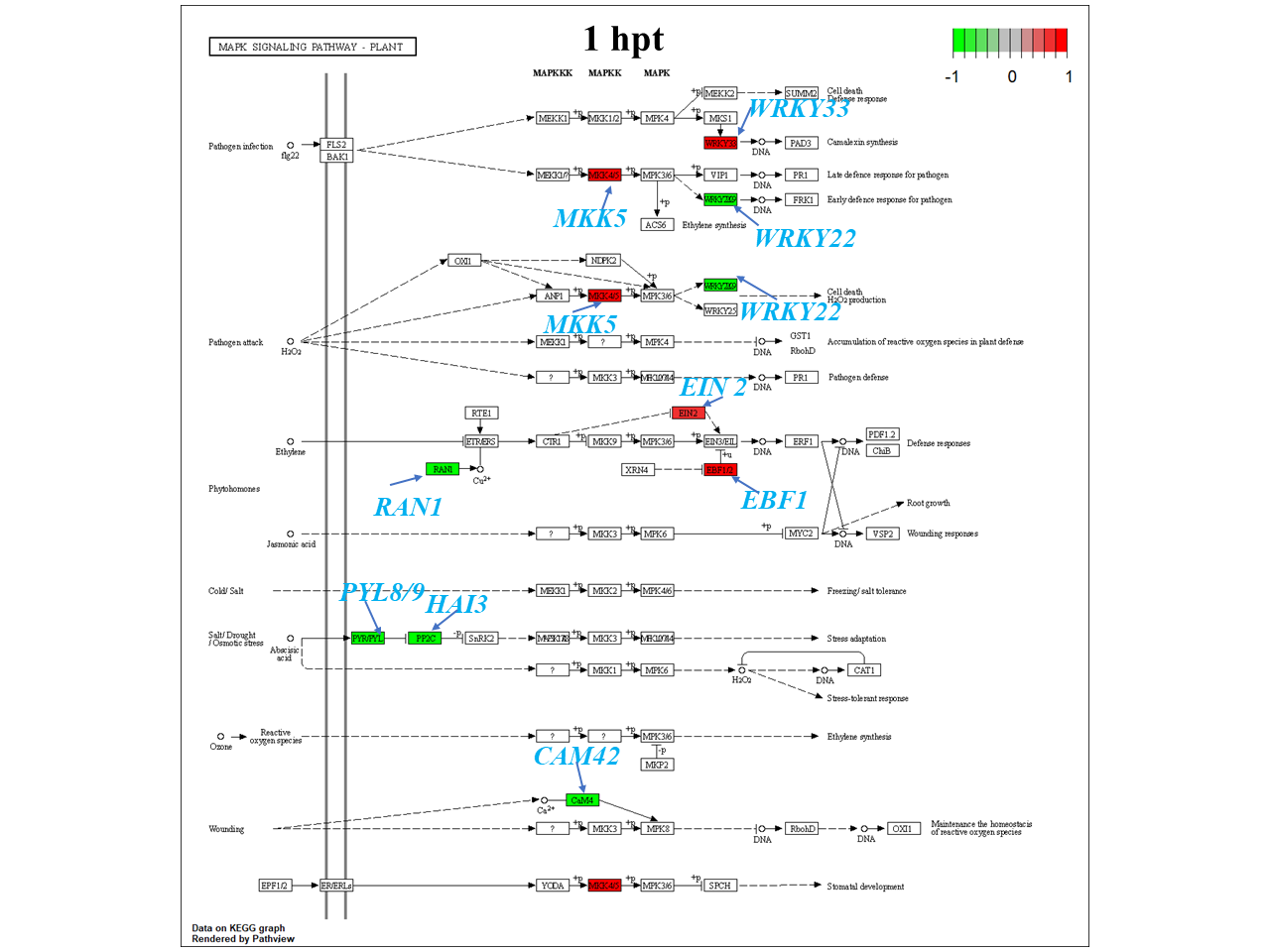

Supplement: S6 Fig — All colored boxes belong to genes annotated in this transcriptome result. Compared to control samples, red boxes represent DEGs with FC >1, and green boxes represent DEGs with FC <1. (TIF) [file pone.0293396.s006.tif]

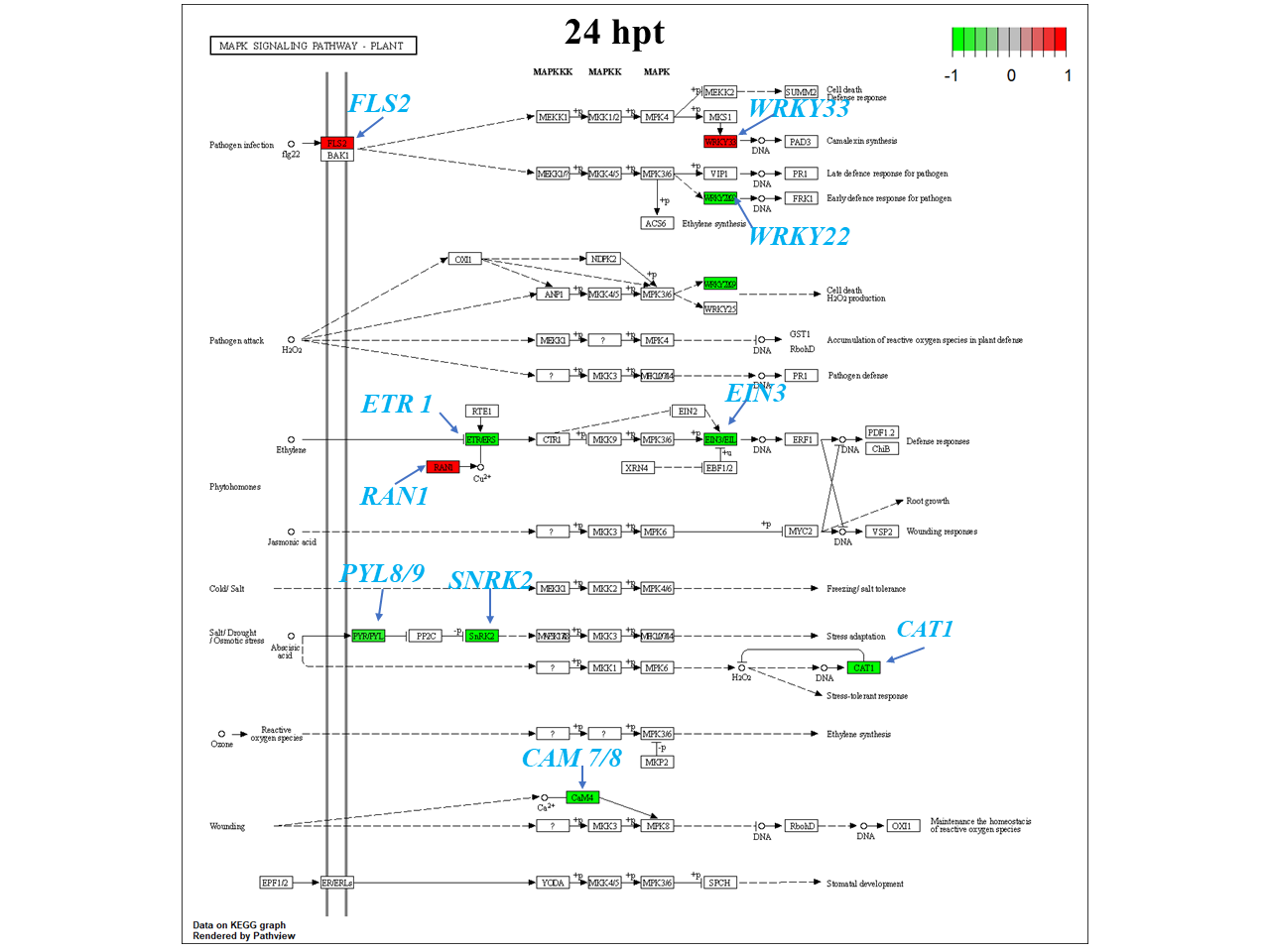

Supplement: S7 Fig — All colored boxes belong to genes annotated in this transcriptome result. Compared to control samples, red boxes represent DEGs with FC >1, and green boxes represent DEGs with FC <1. (TIF) [file pone.0293396.s007.tif]

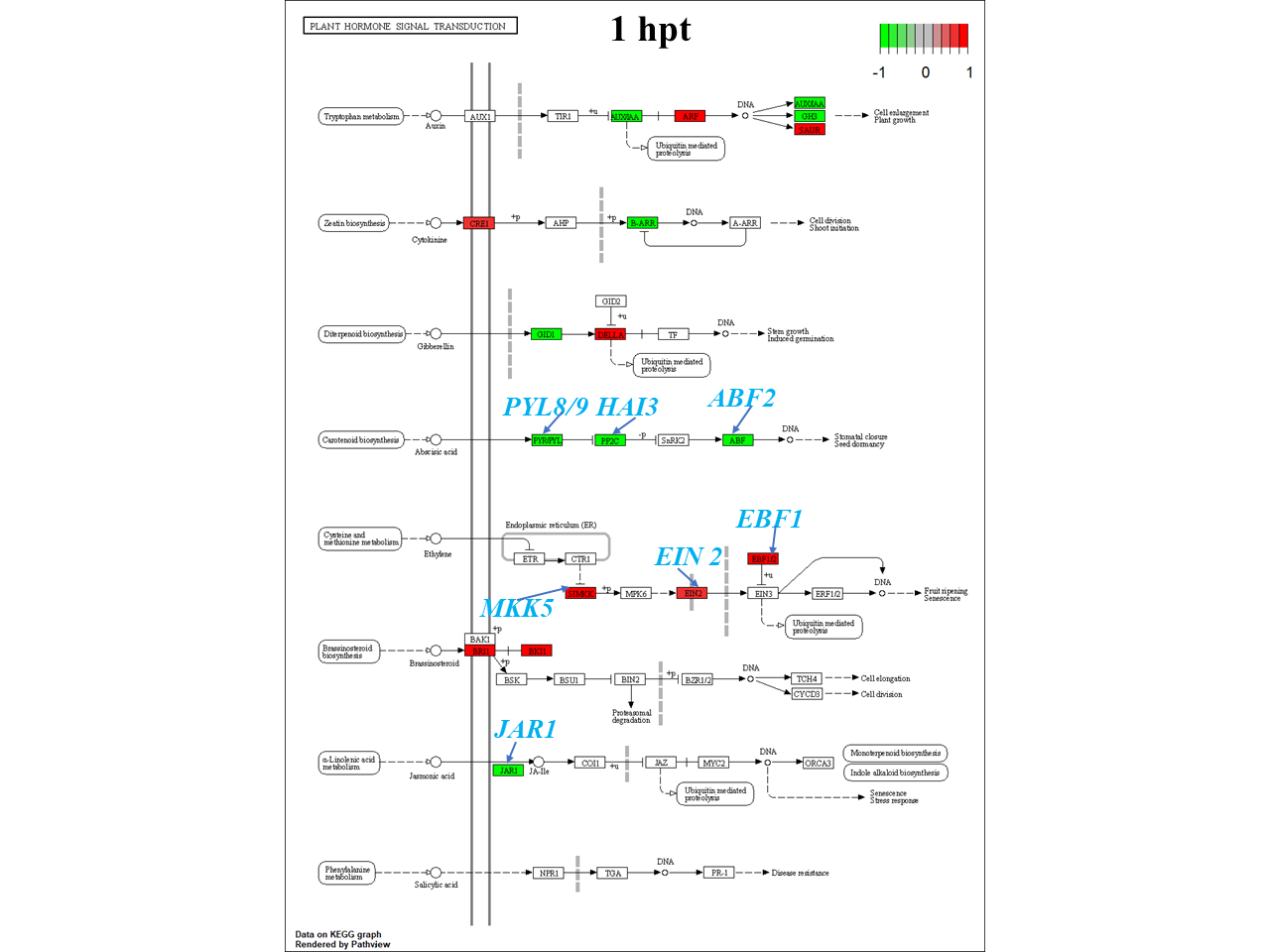

Supplement: S8 Fig — All colored boxes belong to genes annotated in this transcriptome result. Compared to control samples, red boxes represent DEGs with FC >1, and green boxes represent DEGs with FC <1. (TIF) [file pone.0293396.s008.tif]

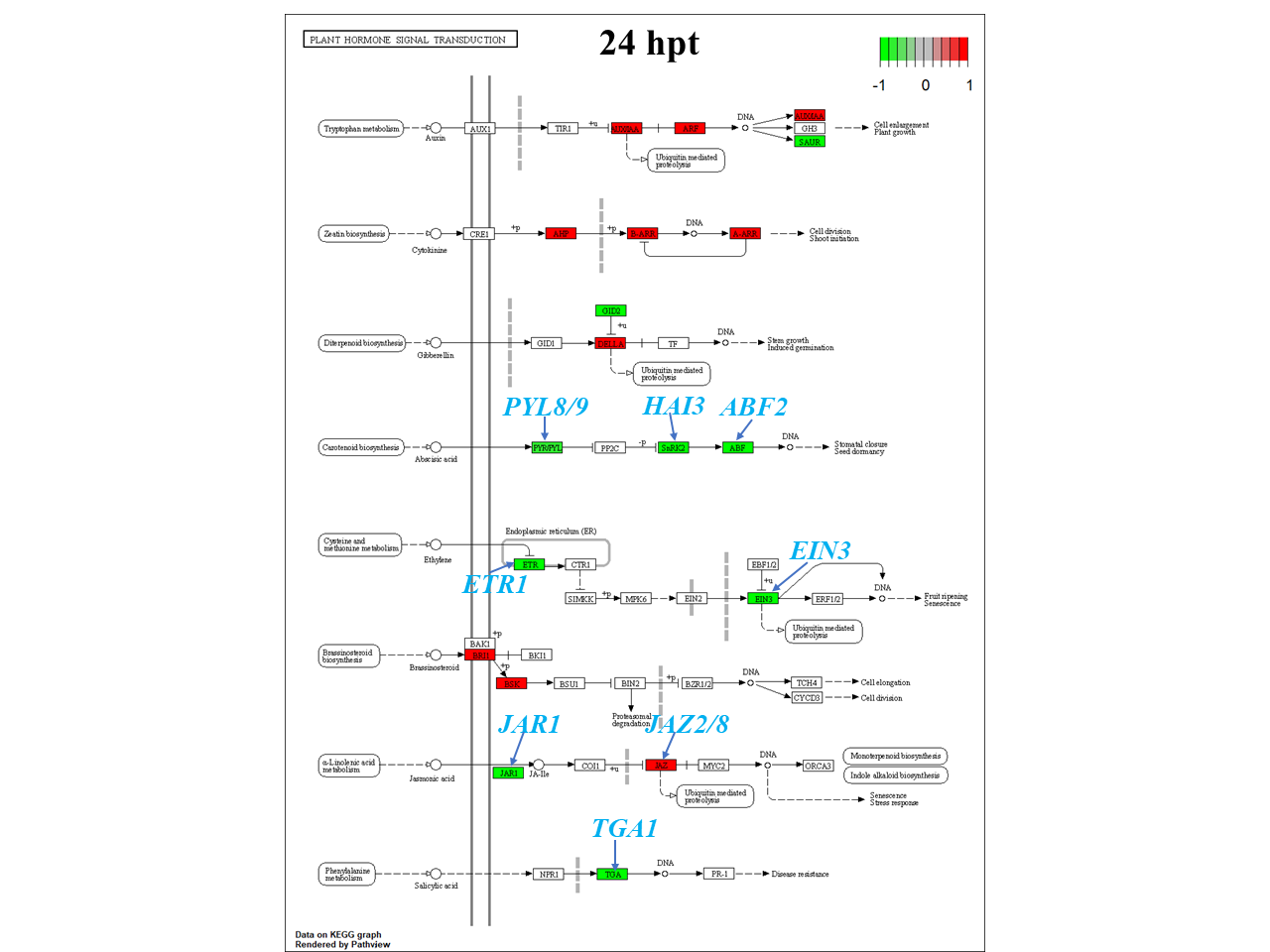

Supplement: S9 Fig — All colored boxes belong to genes annotated in this transcriptome result. Compared to control samples, red boxes represent DEGs with FC >1, and green boxes represent DEGs with FC <1. (TIF) [file pone.0293396.s009.tif]

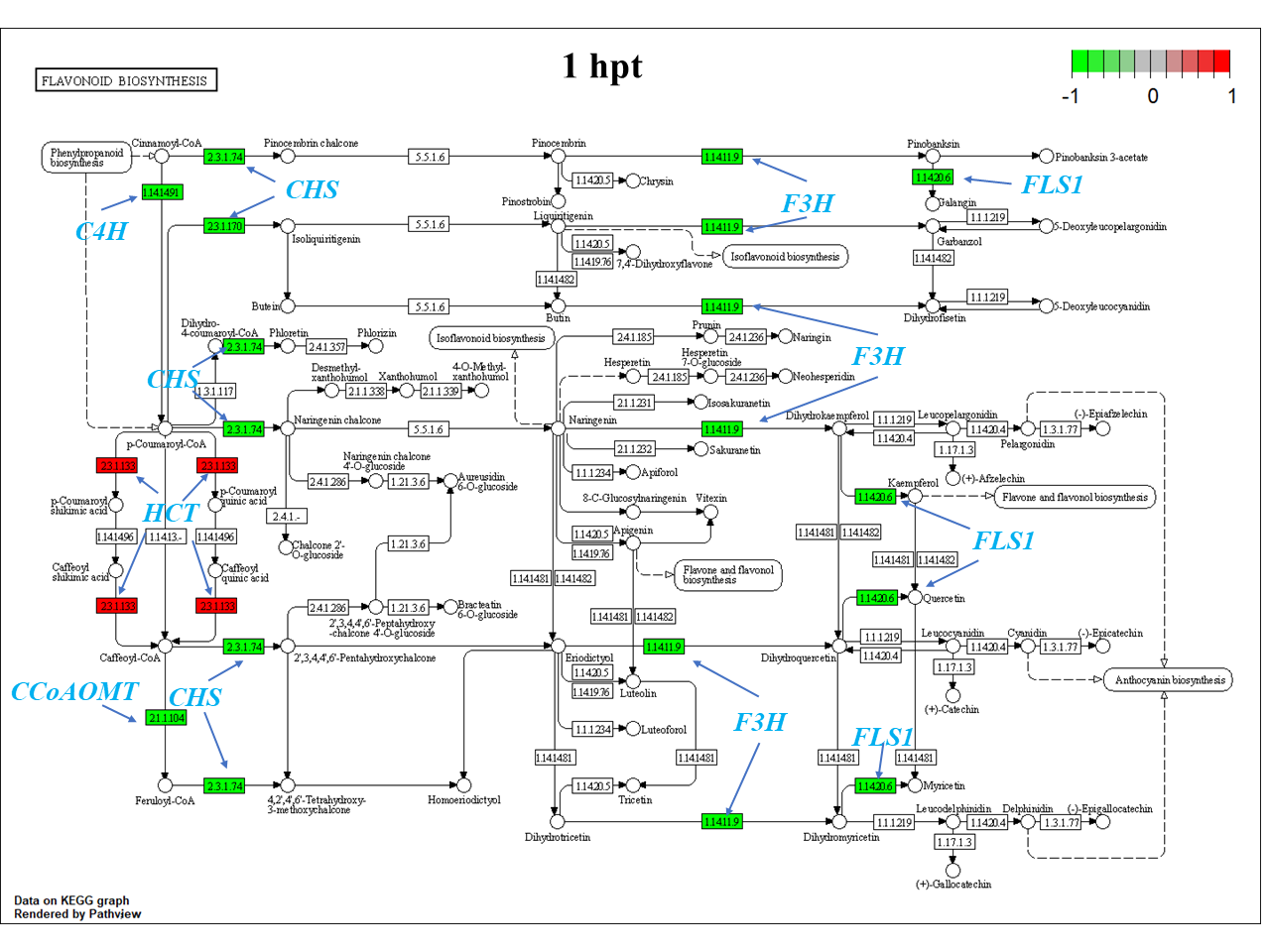

Supplement: S10 Fig — All colored boxes belong to genes annotated in this transcriptome result. Compared to control samples, red boxes represent DEGs with FC >1, and green boxes represent DEGs with FC <1. (TIF) [file pone.0293396.s010.tif]

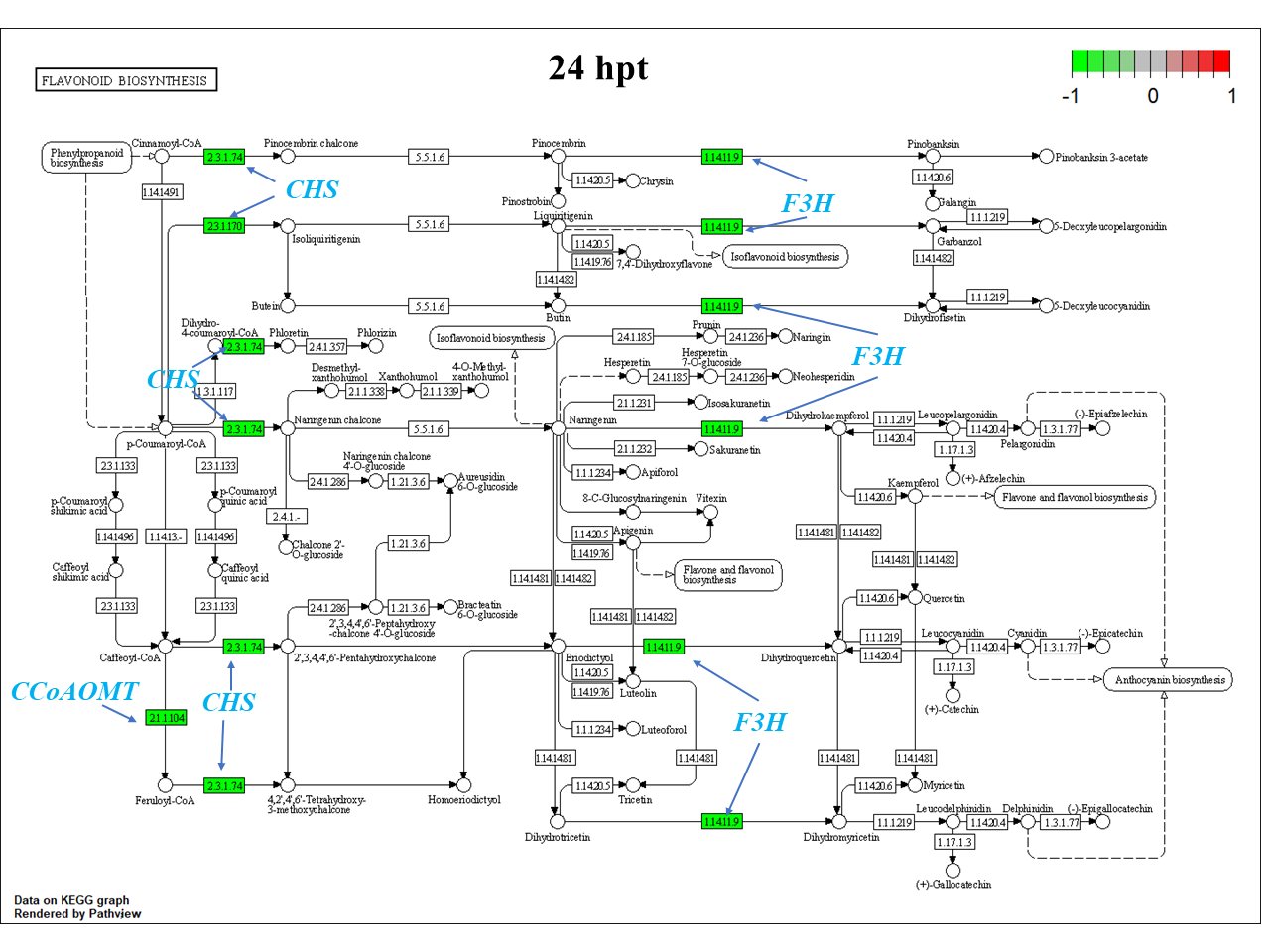

Supplement: S11 Fig — All colored boxes belong to genes annotated in this transcriptome result. Compared to control samples, red boxes represent DEGs with FC >1, and green boxes represent DEGs with FC <1. (TIF) [file pone.0293396.s011.tif]

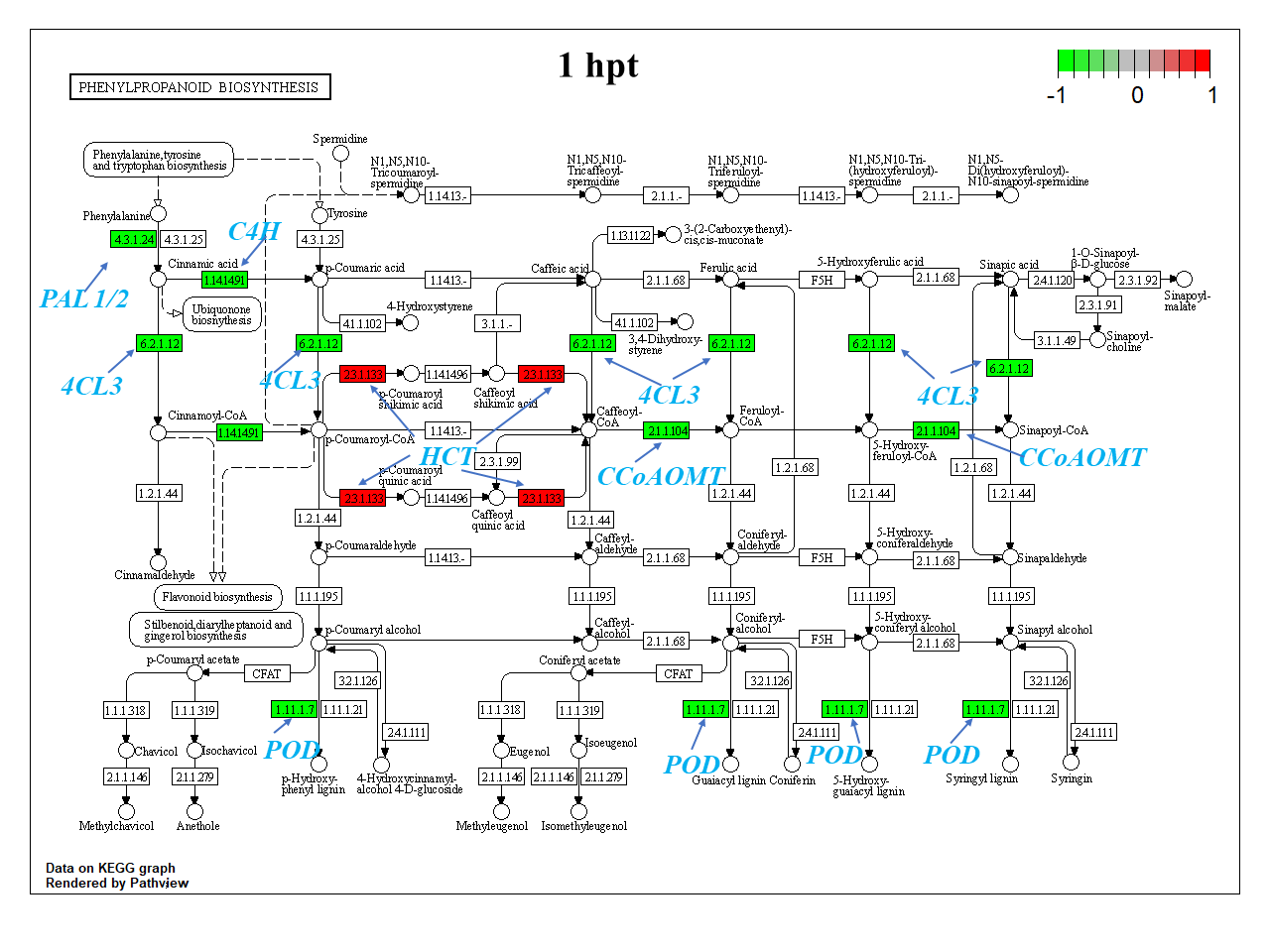

Supplement: S12 Fig — All colored boxes belong to genes annotated in this transcriptome result. Compared to control samples, red boxes represent DEGs with FC >1, and green boxes represent DEGs with FC <1. (TIF) [file pone.0293396.s012.tif]

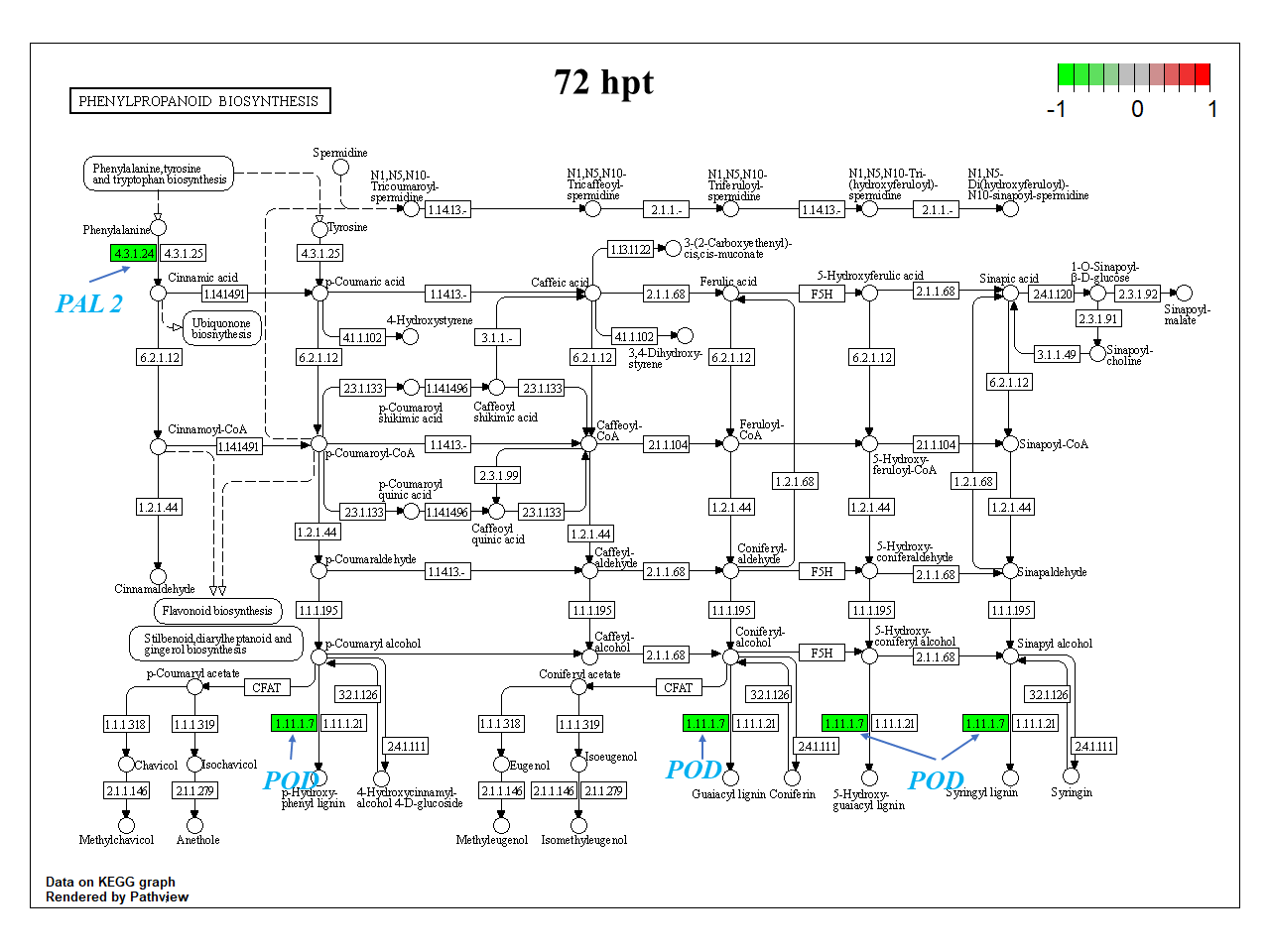

Supplement: S13 Fig — All colored boxes belong to genes annotated in this transcriptome result. Compared to control samples, red boxes represent DEGs with FC >1, and green boxes represent DEGs with FC <1. (TIF) [file pone.0293396.s013.tif]

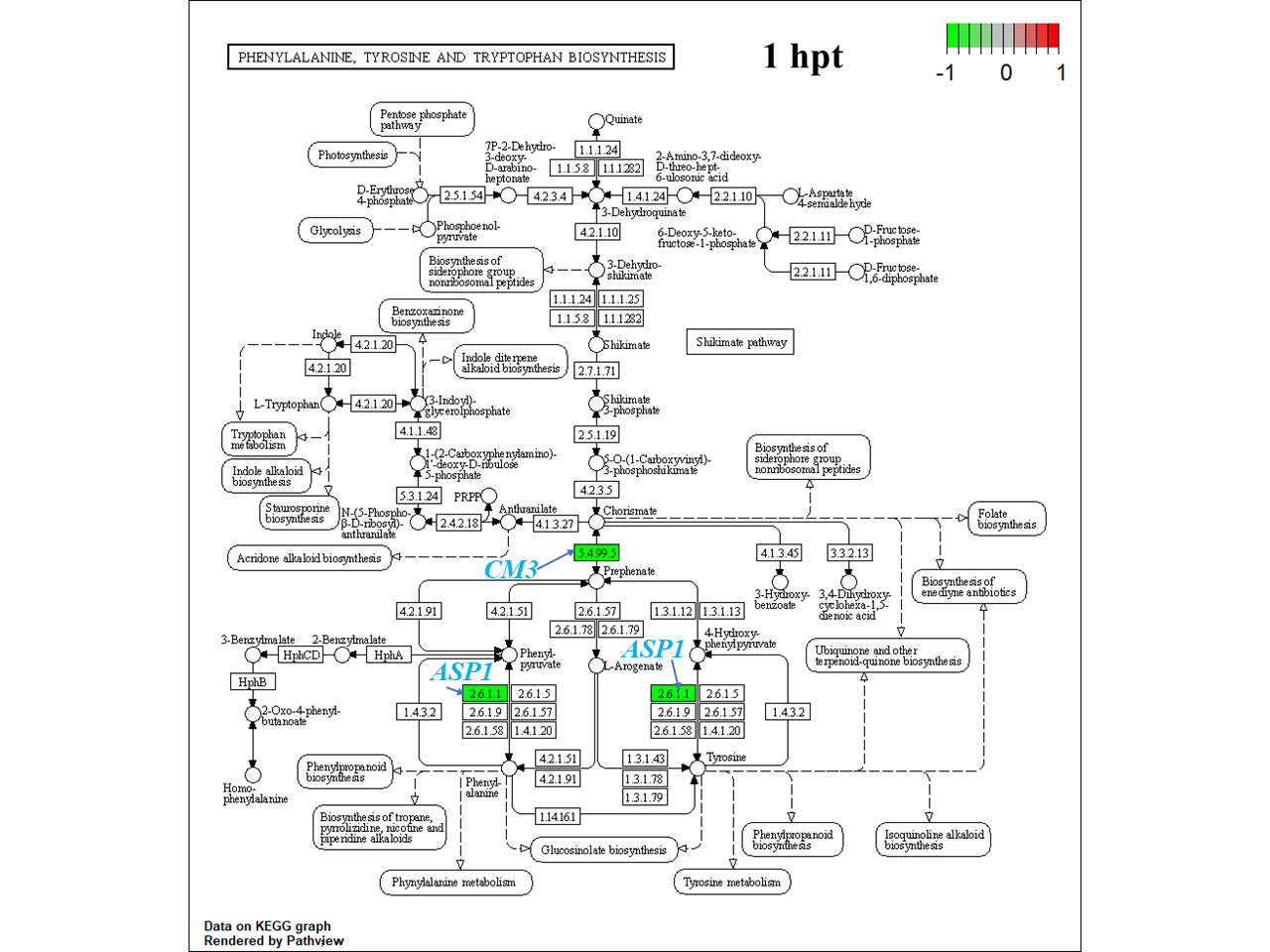

Supplement: S14 Fig — All colored boxes belong to genes annotated in this transcriptome result. Compared to control samples, red boxes represent DEGs with FC >1, and green boxes represent DEGs with FC <1. (TIF) [file pone.0293396.s014.tif]

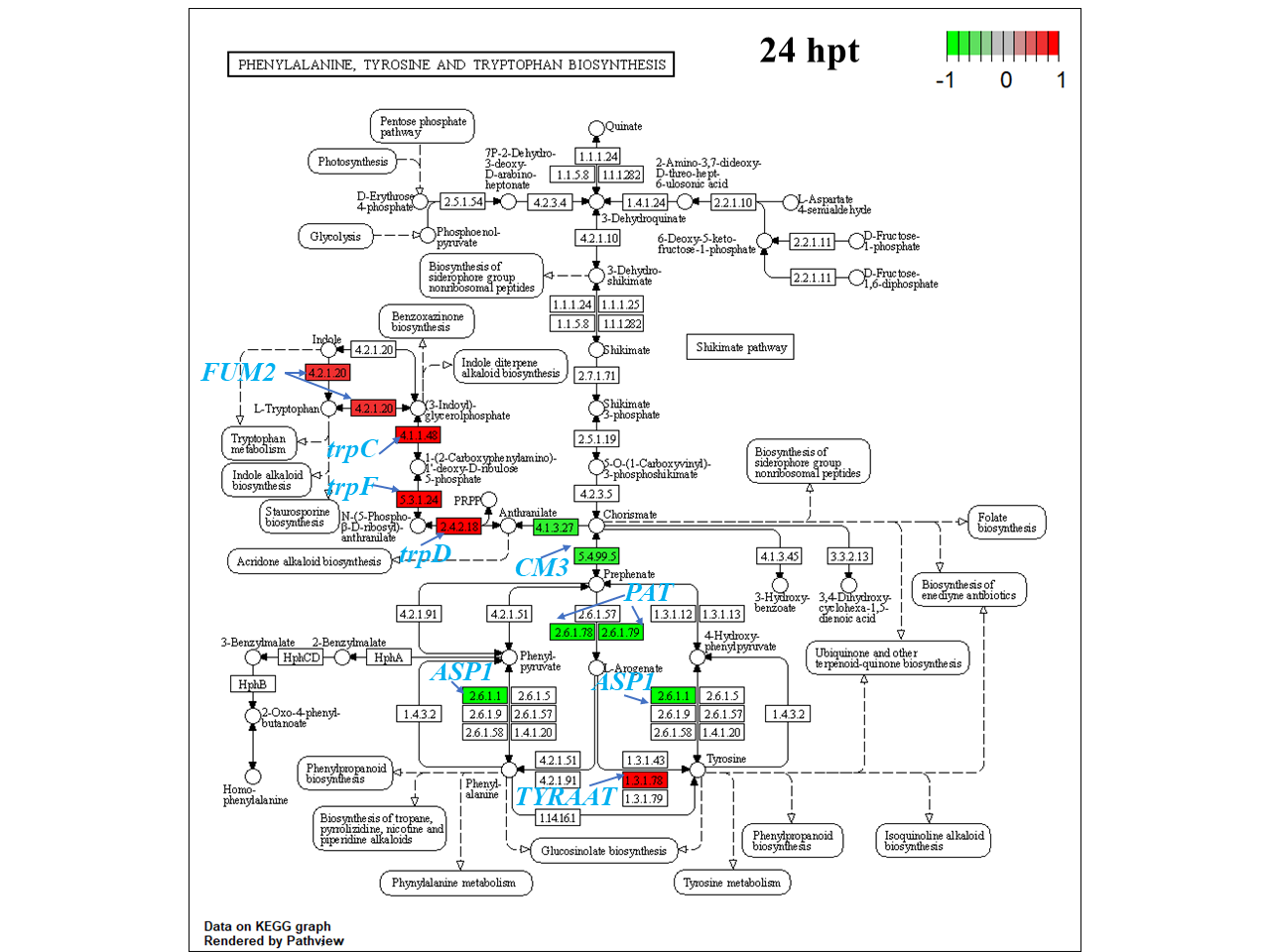

Supplement: S15 Fig — All colored boxes belong to genes annotated in this transcriptome result. Compared to control samples, red boxes represent DEGs with FC >1, and green boxes represent DEGs with FC <1. (TIF) [file pone.0293396.s015.tif]

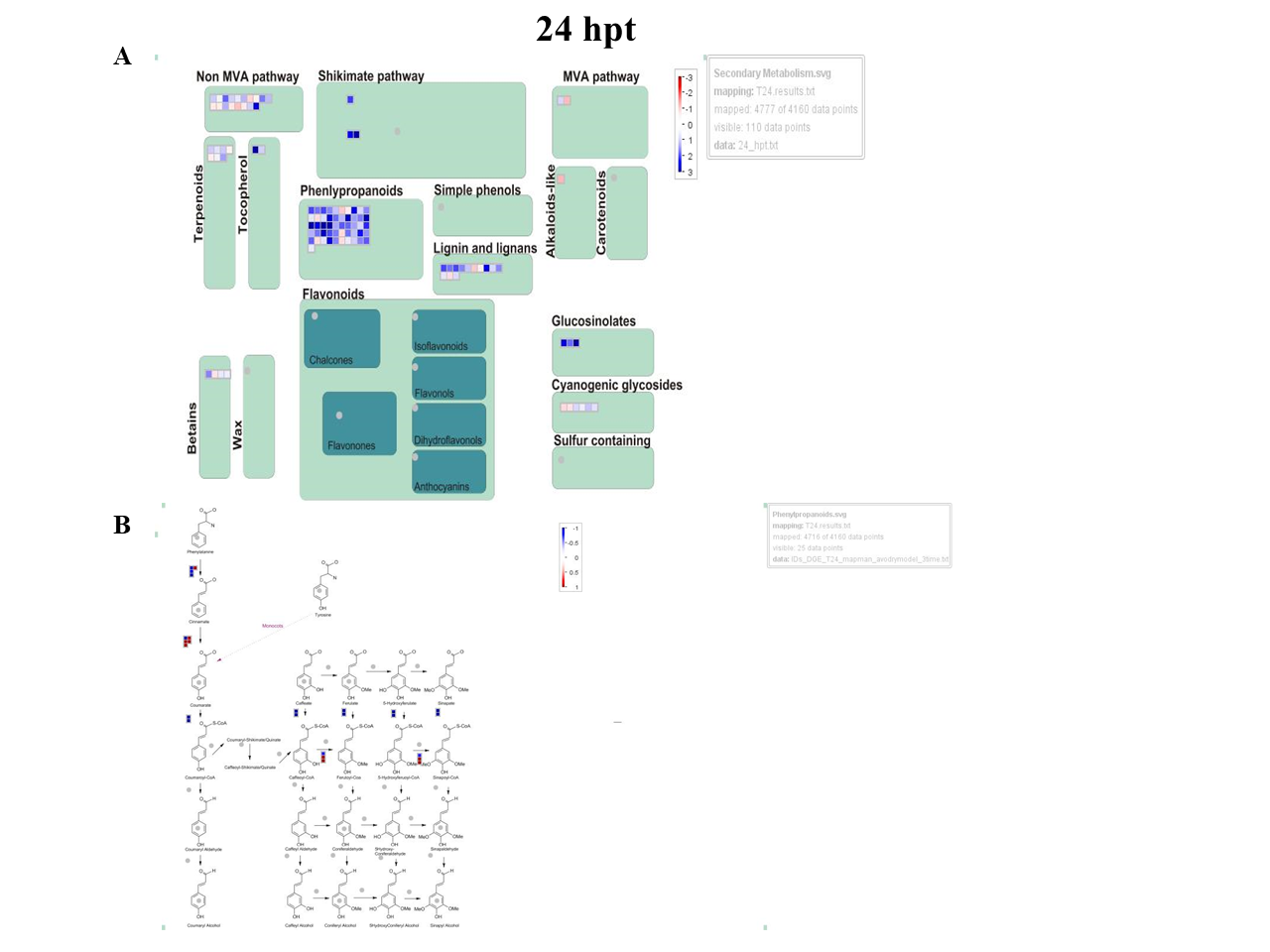

Supplement: S16 Fig — Common responsive genes assigned to secondary metabolism overview (A) and phenylpropanoid biosynthesis (B) based on Mapman software. The secondary metabolism pathway consisted of the genes that participated in the shikimate pathway, the Mevalonate pathway and the biosynthesis of flavonoids, phenylpropanoids, simple phenols and lignin. Different squares indicate DEGs in FCvsFC at 24 hpt comparison, where red indicates up-regulation and blue indicates down-regulation. (TIF) [file pone.0293396.s016.tif]
