# Supplementary material for: Transcriptomic data exploring the effect of agave fructans on the induction of the defense system in avocado fruit
Source: PLoS One. 2023 Oct 26;18(10):e0293396. doi: 10.1371/journal.pone.0293396 (PMC10602311; doi:10.1371/journal.pone.0293396)
Supplement: S1 Table — (PDF) [file pone.0293396.s017.pdf]

S1 Table. Statistics of the transcriptome data

Samples	Raw reads	Clean reads	Data size (Gb)	GC (%)	Total mapped reads	Mapping (%)	Counts (%)
**F1-1**	93818828	88740187	10.4	47	76677139	86.41	82.7
**F1-2**	85597419	81276590	9.2	46	70513584	86.76	81.9
**F1-3**	88528711	83950661	9.8	47	73263002	87.27	80.3
**F24-1**	75361968	71396190	8.3	47	61564482	86.23	82.6
**F24-2**	88251117	83077284	9.8	47	71504027	86.07	84.0
**F24-3**	95191489	90059452	10.4	47	77378523	85.92	81.2
**F72-1**	82754272	78731110	9.2	48	68500332	86.12	83.3
**F72-2**	83758105	79544014	9	47	67964936	86.33	84.2
**F72-3**	86029065	81759210	9.2	47	70273941	85.95	84.9
**FC1-1**	89819288	86448894	11.5	47	91317516	88.36	82.6
**FC1-2**	89782506	86052645	9.6	47	76538073	88.54	82.3
**FC1-3**	72507403	69551937	9.6	47	75543708	87.79	83.1
**FC24-1**	77072011	73152269	8.5	48	62450827	85.37	83.8
**FC24-2**	80980784	76594837	8.2	47	59820547	84.93	84.2
**FC24-3**	83113885	78762634	8.8	48	66055483	86.24	85.6
**FC72-1**	80513958	80513958	9.7	47	72969158	87.01	84.7
**FC72-2**	88520848	83867646	7.9	47	59845389	86.93	85.6
**FC72-3**	89049905	84689614	9.8	47	73574155	86.88	85.0
